# A new multiple image encryption algorithm using hyperchaotic systems, SVD, and modified RC5

**DOI:** 10.1038/s41598-025-92065-x

**Published:** 2025-03-21

**Authors:** Wassim Alexan, Mohamed Youssef, Hisham H. Hussein, Karim K. Ahmed, Khalid M. Hosny, Abdallah Fathy, Marvy Badr Monir Mansour

**Affiliations:** 1 Communications Department, Faculty of Information Engineering and Technology, German University in Cairo (GUC), Cairo, Egypt; 2 Department of Mathematics, Faculty of Engineering, German International University (GIU), New Administrative Capital, Cairo, Egypt; 3 Computer Science Department, Faculty of Media Engineering and Technology, German University in Cairo (GUC), Cairo, Egypt; 4 School of Mathematical and Computational Sciences, University of Prince Edward Island (UPEI), Cairo Campus, The New Administrative Capital, Cairo, Egypt; 5 Department of Information Technology, Zagazig University, Zagazig, Egypt; 6 Department of Electronic Engineering Technology, Elsewedy University of Technology, 10th of Ramadan City, 7060010 Egypt; 7 Department of Electrical Engineering, Faculty of Engineering, The British University in Egypt, 11837 Cairo, Egypt

**Keywords:** Hyperchaos, Image encryption, NIST, Security analysis, SVD, RC5, Engineering, Mathematics and computing

## Abstract

Secure image encryption is critical for protecting sensitive data such as satellite imagery, which is pivotal for national security and environmental monitoring. However, existing encryption methods often face challenges such as vulnerability to traffic analysis, limited randomness, and insufficient resistance to attacks. To address these gaps, this article proposes a novel multiple image encryption (MIE) algorithm that integrates hyperchaotic systems, Singular Value Decomposition (SVD), counter mode RC5, a chaos-based Hill cipher, and a custom S-box generated via a modified Blum Blum Shub (BBS) algorithm. The proposed MIE algorithm begins by merging multiple satellite images into an augmented image, enhancing security against traffic analysis. The encryption process splits the colored image into RGB channels, with each channel undergoing four stages: additive confusion using a memristor hyperchaotic key transformed by SVD, RC5 encryption in counter mode with XOR operations, Hill cipher encryption using a 6D hyperchaotic key and invertible matrices mod 256, and substitution with a custom S-box generated by a modified BBS. Experimental results demonstrate the proposed algorithm’s superior encryption efficiency, enhanced randomness, and strong resistance to cryptanalytic, differential, and brute-force attacks. These findings highlight the MIE algorithm’s potential for securing satellite imagery in real-time applications, ensuring confidentiality and robustness against modern security threats.

## Introduction

The escalating demand for secure transmission and storage of satellite imagery in diverse areas such as national security and environmental monitoring emphasizes the urgent need for sophisticated image encryption techniques^1,2^. Satellite images, by their nature, contain highly sensitive data that, if compromised, could lead to significant breaches in security or privacy. Conventional encryption algorithms such as the Advanced Encryption Standard (AES) often fail to address the unique challenges posed by the high redundancy and substantial data volumes inherent in image files, leading to potential inefficiencies and vulnerabilities^3^. Moreover, the specific threats to satellite imagery, including susceptibility to traffic analysis attacks, necessitate the development of tailored encryption methodologies that ensure robust protection and data integrity across extensive and frequently public networks^4^.

The encryption strategy introduced in this article incorporates a suite of advanced cryptographic techniques, each selected for their complementary strengths in enhancing security. Hyperchaotic systems are utilized for key generation due to their high sensitivity to initial conditions, providing a robust foundation for cryptographic applications^5,6^. Singular Value Decomposition (SVD) is applied to these keys to further fortify the encryption mechanism^7^. The adaptation of RC5 in counter mode accommodates the high data throughput demands of satellite imagery while ensuring dynamic security^8^. Additionally, a chaotic-based Hill cipher introduces spatial transformations that preserve the structural integrity of the images. Lastly, a custom S-box, developed using a modified Blum Blum Shub (BBS) algorithm, injects Non-Linearity (NL) and complexity into the system, significantly enhancing resistance to cryptographic attacks. Each component has been integrated not only for its individual efficacy but also for its synergistic effect on the overall encryption process, ensuring comprehensive security for sensitive satellite images. Importantly, the algorithm is optimized for speed, operating in real-time to support the immediate encryption needs of high-resolution satellite data.

This research work makes several significant contributions to the field of image encryption, particularly for enhancing the security of satellite imagery. First, it introduces a MIE algorithm that integrates the SVD, counter mode RC5, a chaotic-based Hill cipher, and a custom S-box using a modified BBS algorithm, tailored specifically for the complexities of high-dimensional image data. Second, it demonstrates the effectiveness of this algorithm through rigorous numerical testing, showcasing strong resistance to statistical, differential, and brute-force attacks. Additionally, the article proposes a novel method for merging multiple satellite images into a single augmented image, significantly enhancing protection against traffic analysis attacks. Lastly, it validates the encryption performance through extensive cryptographic analyses, setting a new standard for secure image transmission and storage in critical applications like national security and environmental monitoring. This article is organized as follows. Section “Related literature” carries out a review of existing methods and technologies in image encryption. It specifically highlights previous work on hyperchaotic systems, SVD, RC5, Hill ciphers, and custom S-boxes, establishing the foundation and motivation for the novel contributions of this article. Section “Preliminaries” provides the necessary preliminary cryptographic constructs made use of in this research work. Section “Proposed MIE algorithm” gives a description of the proposed satellite image encryption algorithm. Section “Performance evaluation and numerical results” carries out a performance evaluation of the proposed algorithm. Ultimately, Section “Conclusions and suggested future research” draws the conclusions and provides suggestions for future research work.

## Related literature

This literature review covers the use of the foundational techniques that underpin the proposed image encryption algorithm, exploring the integration and adaptation of established cryptographic strategies and advanced mathematical theories. Specifically, the review focuses on four pivotal components: SVD, the RC5 block cipher and its variants, hyperchaotic systems, the Hill cipher and its variants. Each of these components has been instrumental in the development of existing encryption methods, offering unique strengths and potential areas for enhancement. By examining how these techniques have been previously employed and their performance in securing image data, this review sets the stage for the introduction of a novel encryption algorithm that synthesizes these elements to improve security and efficiency in image encryption.

SVD based schemes have proven success in numerous applications in image processing and encryption^9–16^. For instance, the SVD recipe has been constantly applied in image enhancement, reconstruction, and compression, color magnification, actual resolution estimation, color to grayscale image conversion, Joint Photographic Experts Group (JPEG) image steganography, image retrieval, and securing medical images. Primarily, SVD based approaches have been intensively employed in image encryption. For instance, in^9^, a smart asymmetric SVD-based color image encryption technique is proposed. The given image is to be encrypted into a cipher-text shown as an indexed image. The RGB components are then coded into a complex function, which is then separated into U, S, and V via SVD. The cipher-text data matrix is found by multiplying orthogonal matrices U and V while applying phase-truncation. Diagonal entries of the 3 diagonal matrices of the SVD outcomes are abstracted and combined to form the color-map of the cipher-text. In the decryption phase, the original color image is retrieved through private keys, obtained from phase-truncation and the orthogonality of V. In addition, to securing medical images, a novel SVD-based image encryption scheme is proposed in^10^. The scheme is also based on chaotic system and Fractional Discrete Cosine Transform (FrDCT) to strengthen the efficiency and reliability of medical image encryption, demonstrating the benefits of FrDCT over fractional Fourier transform. Moreover, the topology of an encryption method, which suggests a robust and fast security of fingerprint images when they transit in porous transmission channels, is proposed in^11^. Fingerprint images’ pixels are transformed with sequences generated via logistic chaotic maps, before they are dispersed by organized arrangements obtained by hyperchaotic systems. The diffused pixels are then decomposed via SVD. Cryptographic analyses like key and plaintext sensitivity tests, correlation analysis, as well as entropy test on fingerprint images demonstrate that the technique is robust and efficient against cryptographic attacks. The work in^12^ proposes an asymmetric optical image cryptosystem based on biometric keys and the SVD in the Fresnel transform domain. The authors in^13^ propose a smart image cryptosystem through Multi-Resolution-SVD (MR-SVD) and multiple chaotic maps. The encryption procedure begins by realizing the MR-SVD to analyze the original image into the 4 major sub-bands, i.e., approximation (A), horizontal (H), vertical (V), and diagonal (D) sub-bands. The (A) sub-band is chosen to carry out diffusion and permutation, since it provides the most information concerning the image. The permutation of all the 4 sub-bands is performed by using the Baker map. The pixels’ diffusion in the permuted approximation is accomplished via the neighborhood diffusion structure, which utilizes the numerical solution of the chaotic Thomas’ cyclically symmetric attractor. Eventually, the cipher is achieved by combining the 4 (A, H, V, D) partial ciphers by applying the inverse MR-SVD. Most up-to-date image encryption schemes encrypt plain images into meaningless cipher images. Visually, a few of them are susceptible to illegal attacks on some open channels or sharing platforms when being sent. In this concern, the research work in^14^ proposes a novel significant image encryption scheme based on Compressive Sensing (CS) and information-hiding technology, reducing the probability of being attacked by hiding plain images. In that work, the Discrete Wavelet Transform (DWT) is employed to sparse the plain image, followed by confusion on pixel positions. Next, images are compacted and encrypted through CS to construct an intermediate cipher image, where a measurement matrix is generated through a low-dimension complex tent-sine system. To improve the recovery quality, the authors suggest that the intermediate cipher is filled with random numbers along with the compression ratio. This process involves confusing those random numbers to obtain the secret image. Finally, 2D DWT of the carrier image is performed, followed by SVD. In addition, the authors in^15^ propose a color image encryption scheme based on chaotic systems, which combines CS and block-based DNA coding, and SVD to attain an optimum performance to enhance the encrypted image reconstruction quality. The plain image is compressed via CS to attain 3 measurement value matrices, which are then quantized into integer matrices and permuted by the Josephus problem scheme. In^16^, the authors’ proposed methodology aims to improve the security of medical images during transmission across public networks. The method involves 3 main steps: compression, encryption, and embedding to attain the target of preserving the confidentiality and integrity of medical images. Their work utilizes a multi-step process. First, a medical image undergoes compression via SVD to minimize its size and remove any noise. Then, the compressed image is encrypted through the AES scheme to guarantee confidentiality. Eventually, the encrypted image is securely enclosed within a cover image using the Least Significant Bit (LSB) steganography scheme.

Other state-of-the-art works employ the RC5 block cipher to encrypt digital images^8,17–21^. For example, in^8^, the authors introduce a modified RC5 which increases its key space. This is carried out through performing the XOR function on 16 rounds with a new operation that uses 2 keys and 4 states. In^17^, the authors employed RC5 for image encryption and provided optimum values for its design parameters. The work in^18^ proposed an enhanced RC5 algorithm where round keys are calculated using chaos for more security. Whereas the research in^19^ hybridized the DWT with RC4 for partial image encryption while rearranging the rest of image via a shuffling algorithm. Moreover, tha authors of^20^ present a developed RC5 algorithm where 2D chaotic maps are used to compute round keys. Besides that, the Cipher Feedback Mode (CFB) is employed in^20^ to enhance the diffusion property of the proposed algorithm. In^21^, the authors used diverse block cipher modes of operation for RC5 to attain immediate image encryption.

With regards to the use of hyperchaotic systems in image encryption, various works cover their application^22–37^. In^22^, the authors suggested a fractional-order 5D hyperchaotic system of differential equations and periodic state of integer-order. Employing this system, two novel scrambling algorithms, that are Rubik’s cube and an enhanced Arnold transform, in conjunction with a double diffusion algorithm are provided for image encryption. The authors in^23^ demonstrate a cross-channel image encryption approach for colored images, which utilizes a new 2D hyperchaotic hybrid map. Their approach carries out a diagonal permutation of the image pixels among RGB channels. Also, it applies a bi-directional sequential diffusion on pixel values to enhance the security of encrypted images. In^24^, the authors present an image encryption scheme that uses 4D memristive hyperchaos to generate Substitution boxes (S-boxes) for diffusion and Cellular Automata (CA) for confusion. They provide a hardware implementation of their scheme where the memristor-based system is employed to generate chaotic sequences. While in^25^, the authors showcase a 2-step image cryptosystem that adopts a 5D hyperchaotic system and a Fibonacci Q-matrix (FQ-matrix) for gray images. In this cryptosystem, the image pixels are substituted by a 5D hyperchaotic map, while pixel values are altered via a FQ-matrix to achieve confusion and diffusion. The work in^26^ provides a 3-step architecture for RGB image encryption. Their architecture employs in step one a Sine chaotic map, and in step two a 4D hyperchaotic fractional-order Chen map for the S-box generation. In step three, a hybrid DNA coding algorithm is used to increase the key space and provide confusion.

Additionally, in^27^, the authors present a colored image encryption framework comprised of multiple layers. Their proposed framework utilizes a 4D dynamical fractional-order Chen map, Mersenne Twister, OpenSLL, Rule 30 CA and Intel’s Math Kernel Library for the generation of an encryption key and the construction of an S-box in each layer. Both diffusion and confusion are provided in this framework for more security. Moreover, in^28^, the authors demonstrate an image encryption architecture which combines the chaotic differential systems of fractional-order that are Chen system, Chua system and Memristor system to develop pseudo-random numbers for encryption keys and S-boxes. Also, variable base-*n* S-boxes are used simultaneously to achieve confusion. Furthermore, multiple logical and arithmetic functions are adopted to guarantee data diffusion. The work in^29^ provides an image cryptosystem that hybridizes image transformation methods with chaotic and hyper-chaotic systems. The algorithm implements randomization, rotation, and rescaling on the plain image through the hyperchaotic Chen as well as Chua mathematical models. The initial conditions of chaotic functions utilized enrich this system with a vast key space. Whereas, the authors in^30^ showcase a method for image encryption that consists of three sequential stages and includes two hyperchaotic maps, for obtaining a large key space, and the Single Neuron Model (SNM). The hyperchaotic maps and the SNM are numerically solved and their solutions are used to derive both the Pseudo-Random Number Generator (PRNG) encryption keys and the S-boxes. The proposed method entails in each stage an S-box application, then the XOR function between generated encryption key and image bits. In^31^, the authors proposed a colored image encryption approach integrating the 1D and multiple-dimensional chaotic functions with the KAA map. It grants both confusion and diffusion for improved security. Confusion is attained through two cryptographic keys, where one key is obtained via the Logistic 2D Sine map and the linear congruential generator, whereas the other key is attained using the Tent and Bernoulli maps. On the other hand, data diffusion is ensured via the KAA map adoption. Besides that, in^32^, the authors presented a 3-phase image encryption architecture that employs a 4D hyperchaotic fractional-order Chen map. In the first phase, the authors numerically solve the Chen system to generate its solution and then apply to it the Discrete Fourier Transform (DFT), which is then transformed to be employed in DNA coding. While, in the second phase, an S-box is obtained and utilized via the transformed DFT. In the third phase, a PRNG encryption key is derived via the Mersenne Twister, then transformed to base-$\phi$ followed by a modulo function.

Furthermore, the authors of^33^ introduce a voltage-controlled memristor featuring a fourth-power term, which serves as the basis for a new four-dimensional chaotic system. Their proposed system implemented both in an analog and a digital manner on an FPGA, and applied, along with DNA encoding operations, in an image encryption system. In^34^, a chaos-based image cryptosystem for IIoT is developed, leveraging graph data structures (GDS) and three-input majority gates (TIMG) for secure and flexible image encryption. It introduces a nonsequential diffusion path using breadth-first search and employs TIMG for nonlinear diffusion. Fractional-order discrete maps further enhance key stream flexibility, addressing limitations of traditional diffusion methods. The authors of^35^ propose a tunable memristor model with adjustable multistable states by modifying memory parameters. Using this model, an asymmetric memristive FN-HNN neural network (MFNHNN) with five neurons is constructed and shown to behave in a chaotic manner. An equivalent circuit is implemented, and an image encryption scheme combining Arnold mapping and diagonal diffusion is developed. The work in^36^ proposes a novel multi-channel image encryption algorithm, MIEA-PRHM, which combines pixel reorganization and hyperchaotic maps. Using two hyperchaotic maps, the algorithm generates highly random chaotic sequences with an expanded key space. Input images are first converted into two fused matrices through pixel reorganization. The high 4-bit matrix undergoes two rounds of scrambling and diffusion, plus one substitution round, while the low 4-bit matrix undergoes one substitution and diffusion round. The study in^37^ introduces a robust hyperchaotic map, 2D-SQPM, and develops an efficient image encryption algorithm (IEASP) based on it, incorporating a pixel fusion strategy. IEASP includes several optimizations: a common keystream eliminates the need for frequent key changes, while pixel fusion reduces computational overhead. Additionally, two rounds of vector-level filtering, chaotic pixel superposition, and quick intra-vector scrambling enhance encryption speed and security.

The Hill cipher has also had its fair share of utilization in the literature on image encryption^38–43^. To provide more protection for the image data, the authors in^38^ suggest a combination of the Modified Elliptic Curve Cryptography (MECC) and Hill cipher. Here, ECC represents an asymmetric key encryption and is enhanced further via symmetric encryption of the Hill cipher tolerating fast and simple computations over complex encryption schemes of ECC. Hill cipher encryption involves multiplication of a $4 \times 4$ key matrix with $4 \times 4$ portions of image pixels in which the self-invertible key matrix is obtained from the parameters of the elliptic curve, facilitating and accelerating the decryption process without the need to compute the matrix inverse. In addition, the authors in^39^ suggest a smart color image encryption method, in which after transforming of the original image into a vector and decomposing it into blocks of 3 pixels, along with modifying a seed block by an initialization vector, computed through the plain image, a preliminary confusion is carried out by a substitution matrix developed under the control of the 2 employed chaotic maps. Simulations applied to a large number of color images prove the robustness of the proposed approach against known attacks. Moreover, the authors in^40^ propose a hybrid asymmetric image encryption scheme via ECC, the Hill cipher, and Hadamard transform algorithms. Furthermore, the research work in^41^ proposes a smart color image encryption method based on symmetric keys using the synergistic approach of affine Hill cipher technique, 3D logistic chaotic map with XOR operation, and Arnold transform. All these methods work together and generate a strong cipher to prevent illegal access to data. Besides, the authors in^42^ present an image encryption technique that combines the Kronecker XOR product, Hill cipher, and Sigmoid Logistic Map. The proposed methodology starts by shifting the values in each row of the state matrix to the left by a predetermined number of locations, then encrypting the subsequent image via the Hill cipher. The uppermost value in every even/odd column is used to execute an XOR operation with all values in the corresponding odd/even column, excluding the top value. The results are benchmarked with other literature and are found to have superior performance in terms of differential attack analysis, statistical analysis, Information Entropy (IE) analysis, and brute force attack analysis. In addition, the authors in^43^ introduce a smart image encryption recipe based on a 6D hyperchaotic scheme and the Hill cipher system. Here, the method utilizes the number 257 as a modulo, in which all-zero pixels are exchanged by pixels having a value of 256. Firstly, the original image is divided into 4 equivalent portions to process each part separately. Then, each part is divided into several blocks, each comprising 4 pixels. Secondly, 4 variables of the hyperchaotic arrangement are employed to apply the permutation process on the blocks, in which every variable is used to permute a single part. Thirdly, the 2 enduring variables of the hyperchaotic structure are utilized to produce the Hill matrices. Eventually, each block of each part is encrypted by the Hill cipher using a Hill matrix to obtain the final cipher image.

Table 1 categorizes the reviewed literature into the four components, as well as their combinations. The main problems with current image encryption algorithms, particularly in the context of satellite imagery, include:

1. Vulnerability to traffic analysis attacks: Existing encryption schemes often fail to adequately protect against traffic analysis attacks, where patterns in transmitted data (e.g., redundancy or structure in image data) can be exploited. This is especially critical for satellite imagery, which often contains highly sensitive information.
2. High redundancy in image data: Conventional encryption methods such as AES or DES are not optimized for the inherent high redundancy present in images, which can lead to inefficiencies in encryption and make these methods more susceptible to statistical attacks based on pixel distribution.
3. Insufficient resistance to cryptanalytic attacks: Traditional algorithms often lack sufficient non-linearity or randomness to resist advanced cryptanalytic techniques, such as differential and linear cryptanalysis, which can compromise encrypted image data.
4. Inability to handle real-time processing: Many existing methods are computationally intensive and fail to meet the real-time encryption demands required for high-throughput systems, such as satellite image processing pipelines.
5. Limited key space: Some algorithms suffer from limited key space sizes, making them more vulnerable to brute-force attacks, especially as computational power continues to grow.
6. Lack of robustness against noise and data loss: Satellite images often contain noise or may experience data loss during transmission. Many existing encryption methods are not designed to handle such scenarios effectively, leading to possible decryption errors.

The proposed MIE algorithm addresses these issues as follows:

1. Increased security via hyperchaotic systems: Hyperchaotic systems are used for key generation due to their extreme sensitivity to initial conditions, high-dimensional chaos, and large key space. This enhances the resistance to cryptanalytic attacks and increases randomness.
2. Improved resistance to traffic analysis: By merging multiple satellite images into an augmented image, the proposed method obfuscates traffic patterns and prevents attackers from deducing sensitive information based on transmitted data.
3. Integration of complementary techniques: The inclusion of SVD, counter mode RC5, a chaotic-based Hill cipher, and a custom S-box (via a modified Blum Blum Shub algorithm) ensures a synergistic combination of diffusion, confusion, and non-linearity. These features significantly strengthen the encryption process against statistical and differential attacks.
4. Efficient real-time performance: The proposed MIE algorithm is designed for computational efficiency, leveraging modular operations and parallel processing. This makes it suitable for real-time encryption of high-resolution satellite images.
5. Large key space: The proposed method provides an exceptionally large key space (approximately $2^{10524}$), making brute-force attacks computationally infeasible.
6. Noise tolerance: The preprocessing step includes advanced filtering techniques to handle noise in satellite images, ensuring robust encryption and accurate decryption even in challenging scenarios.

The proposed MIE algorithm is specifically designed to overcome the limitations of existing approaches by addressing their vulnerabilities and optimizing performance for satellite imagery encryption. This ensures enhanced security, efficiency, and robustness in real-world applications such as national security and environmental monitoring.

**Table 1:** Reviewed recent literature categorization.

Ref.	Method				
	Chaos theory	SVD	RC5 & variants	Hill cipher	Goal
^8^			$\checkmark$		KS, E, RRA
^9^		$\checkmark$			E
^10^	$\checkmark$	$\checkmark$			E
^11^	$\checkmark$	$\checkmark$			RRA
^12^	$\checkmark$	$\checkmark$			RRA
^13^	$\checkmark$	$\checkmark$			RRA
^14^		$\checkmark$			RRA
^15^	$\checkmark$	$\checkmark$			RRA
^16^		$\checkmark$			E, RRA
^17^			$\checkmark$		E
^18^	$\checkmark$		$\checkmark$		E, RRA
^19^			$\checkmark$		E, RRA
^20^	$\checkmark$		$\checkmark$		E, RRA
^21^			$\checkmark$		KS, E, RRA
^22^	$\checkmark$				E, RRA
^23^	$\checkmark$				E, RRA
^24^	$\checkmark$				E, RRA
^25^	$\checkmark$				RRA
^26^	$\checkmark$				KS, E, RRA
^27^	$\checkmark$				KS, E, RRA
^28^	$\checkmark$				KS, E, RRA
^29^	$\checkmark$				KS, E, RRA
^30^	$\checkmark$				KS, E, RRA
^31^	$\checkmark$				E, RRA
^32^	$\checkmark$				KS, E, RRA
^33^	$\checkmark$				KS, E, RRA
^34^	$\checkmark$				E, RRA
^35^	$\checkmark$				KS, RRA
^36^	$\checkmark$				KS, E, RRA
^37^	$\checkmark$				KS, E, RRA
^38^				$\checkmark$	RRA
^39^	$\checkmark$			$\checkmark$	RRA
^40^				$\checkmark$	KS, E, RRA
^41^	$\checkmark$			$\checkmark$	RRA
^42^				$\checkmark$	RRA
^43^	$\checkmark$			$\checkmark$	RRA
^44^	$\checkmark$				RRA

In the *Goal* column, the acronyms KS, E, and RRA are used, representing key space > $2^{500}$, efficiency, and robustness and resistance to attacks, respectively.

## Preliminaries

In this section, the foundational elements of the proposed MIE algorithm are presented and analyzed. A variety of mathematical, cryptographic, and chaotic systems are introduced, each selected for its unique properties that enhance the proposed MIE algorithm’s robustness, efficiency, and security. Techniques such as additive confusion for pattern obfuscation, SVD for matrix optimization, the RC5 block cipher for lightweight encryption, and counter mode encryption for stream-based operations are discussed. Hyperchaotic systems, including the memristive and 6D hyperchaotic systems, are explored for their unpredictability, while classical methods like the Hill cipher and modern primitives such as the Blum Blum Shub PRNG are examined to further ensure security. Together, these components are integrated to achieve high-security guarantees with computational efficiency.

### Additive confusion

Additive confusion is a technique used in cryptographic systems to confuse the characteristics of image data across the encrypted output. This method involves modular addition, making it an effective approach for obscuring patterns in the original image^45^. To encrypt an image, each pixel value $p_{i,j}$ is combined with a corresponding key stream value $k_{i,j}$ using modular addition. The mathematical representation of this operation for each pixel can be expressed as:

1 $$\begin{aligned} c_{i,j} = (p_{i,j} + k_{i,j}) \mod n, \end{aligned}$$

where $c_{i,j}$ is the encrypted pixel value, $p_{i,j}$ is the original pixel value, $k_{i,j}$ is the key stream value, and $n$ is the modulus, typically 256 for 8-bit image data. This operation ensures that even small changes in the pixel values result in substantial and unpredictable changes in the encrypted image.

Decryption reverses the encryption process using modular subtraction. For a particular key stream, the values of the original pixel are retrieved by subtracting the key stream from the values of the encrypted pixel:

2 $$\begin{aligned} p_{i,j} = (c_{i,j} - k_{i,j}) \mod n \end{aligned}$$

The primary advantage of additive confusion in image encryption is its efficiency and simplicity, making it suitable for rapid processing in both software and hardware. By linearly dispersing the characteristics of the original image across the encrypted output, it helps prevent statistical attacks based on frequency analysis of pixel values. For maximum security, it is crucial that the key stream be highly random and ideally used only once, similar to a one-time pad. In practical applications, combining additive confusion with other cryptographic techniques, such as substitution and transposition, can significantly enhance the security of an image encryption system^46^.

### SVD

A key matrix factorisation method in linear algebra, SVD has many uses, especially in relation to cryptography^7,9–16^. It decomposes any $m\times n$ matrix *A* into the form

3 $$\begin{aligned} A=U\Sigma V^T, \end{aligned}$$

where *U* represents an $m\times m$ orthogonal matrix containing the left singular vectors, *V* is an $n\times n$ orthogonal matrix containing the right singular vectors, and $\Sigma$ is an $m\times n$ diagonal matrix with the singular values of *A* on its diagonal. These singular values have a decreasing order of magnitude and are non-negative. Due to its ability to simplify and analyse the structure of matrices representing linear transformations in cryptosystems, the SVD is very important in cryptographic applications. This helps with tasks like safe key creation, encryption algorithm optimisation, and cryptanalysis^47^. It is an effective tool for boosting the security and effectiveness of cryptographic techniques due to its capacity to disclose the intrinsic characteristics of a matrix^48^.

A numerical example is provided next. Consider the following $4\times 5$ matrix:

4 $$\begin{aligned} M=\begin{bmatrix} 1 & 0 & 0& 0 & 2 \\ 0 & 0 & 3& 0& 0 \\ 0 & 0 & 0& 0 & 0 \\ 0 & 2 & 0& 0 & 0 \\ \end{bmatrix}. \end{aligned}$$

An SVD of this matrix is given by $U\Sigma V^T$, where

5 $$\begin{aligned} U=\begin{bmatrix} 0 & 1 & 0& 0 \\ -1 & 0 & 0& 0\\ 0 & 0 & 0& 1 \\ 0 & 0 & -1& 0 \\ \end{bmatrix}, \end{aligned}$$

      

6 $$\begin{aligned} \Sigma =\begin{bmatrix} 3 & 0 & 0& 0& 0 \\ 0 & \sqrt{5} & 0& 0& 0\\ 0 & 0 & 2& 0& 0 \\ 0 & 0 & 0& 0& 0 \\ \end{bmatrix}, \end{aligned}$$

7 $$\begin{aligned} V^T=\begin{bmatrix} 0 & 0 & -1 & 0& 0 \\ -\sqrt{0.2} & 0 & 0& 0& -\sqrt{0.8}\\ 0 & -1 & 0& 0& 0 \\ 0 & 0 & 0& 1& 0 \\ -\sqrt{0.8} & 0 & 0& 0& \sqrt{0.2} \\ \end{bmatrix}. \end{aligned}$$

The scaling matrix $\Sigma$ is zero outside of the diagonal and one diagonal element is zero. Furthermore, because the matrices *U* and $V^T$ are unitary, multiplying by their respective conjugate transposes yields identity matrices, as in (8) and in (9). In this case, because *U* and $V^T$ are real-valued, each is an orthogonal matrix.

8 $$\begin{aligned} UU^T=\begin{bmatrix} 1 & 0 & 0& 0 \\ 0 & 1 & 0& 0\\ 0 & 0 & 1& 0 \\ 0 & 0 & 0& 1 \\ \end{bmatrix}=I_4, \end{aligned}$$

9 $$\begin{aligned} VV^T=\begin{bmatrix} 1 & 0 & 0& 0 & 0\\ 0 & 1 & 0& 0& 0\\ 0 & 0 & 1& 0 & 0\\ 0 & 0 & 0& 1 & 0\\ 0 & 0 & 0& 0 & 1\\ \end{bmatrix}=I_5. \end{aligned}$$

This particular SVD is not unique. Choosing *V* such that

10 $$\begin{aligned} V^T=\begin{bmatrix} 0 & 1 & 0 & 0 & 0\\ 0 & 0 & 1 & 0 & 0\\ \sqrt{0.2} & 0 & 0 & 0 & \sqrt{0.8}\\ \sqrt{0.4} & 0 & 0 & \sqrt{0.5} & -\sqrt{0.1}\\ -\sqrt{0.4} & 0 & 0 & \sqrt{0.5} & \sqrt{0.1}\\ \end{bmatrix} \end{aligned}$$

is also a valid SVD.

### RC5

RC5 represents a symmetric block cipher key, which is known for its efficiency and simplicity. Developed by Ronald Rivest in 1994, it is characterized by its flexibility, allowing users to parameterize the number of rounds, block size, and key length. The cipher typically operates on small block sizes that can be 32, 64, or 128 bits, making it adaptable for various encryption needs^49^.

**Fig. 1:**
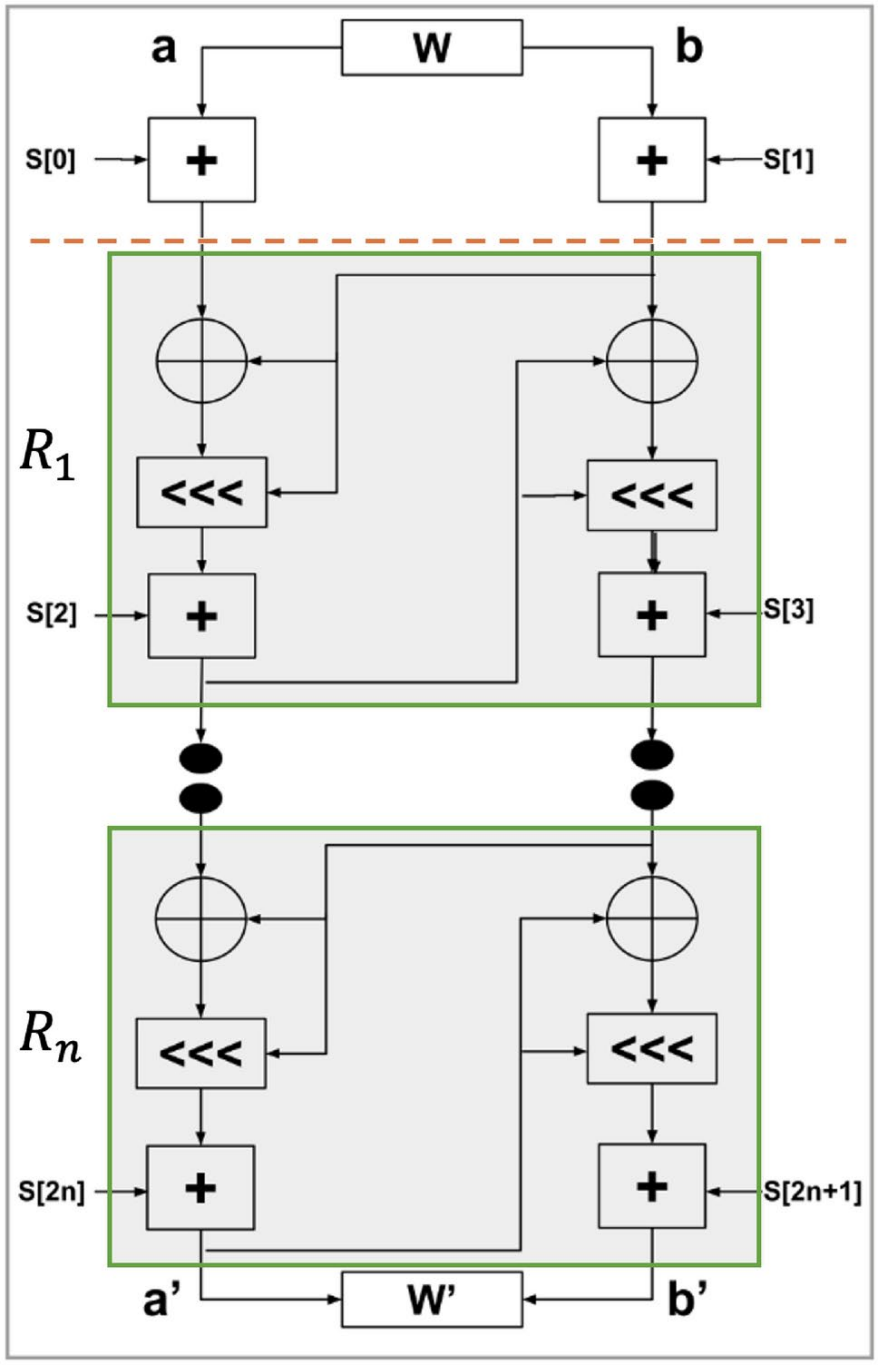
RC5 encryption algorithm.

The core of the RC5 encryption algorithm is described in Fig. 1. It involves a combination of operations including addition, XOR, and variable data-dependent rotations, which are crucial for its high security and performance. Each turn of the cipher combines the key material and plaintext through these operations. The key is extended into a superior array, utilized in the round functions to generate the eventual encrypted image. This straightforward structure facilitates rapid encryption and decryption processes.

RC5’s design allows for adjustments between security and performance, enabling users to customize the cipher’s strength according to specific security requirements and operational contexts. The cipher’s adaptability, ease of implementation, and resistance to cryptanalysis have made it a favored choice in cryptographic applications where both security and performance are paramount^50^.

### Counter mode encryption

Counter-mode encryption converts a block-cipher into a stream-cipher by mixing a “counter” sequence and a fixed key to produce a bit-stream, which is then XORed with plaintext to create ciphertext^51^. This mode’s simplicity allows for the parallel processing of blocks, enhancing speed in large data volume environments or where rapid processing is essential. CTR mode maintains the security level of the block cipher, ensures minimal impact from transmission errors since only the affected bits are corrupted, and supports random access to encrypted data blocks, beneficial for database encryption or file systems. Precomputing the encryption of counter values can further reduce latency, making CTR mode an efficient and secure choice for symmetric encryption, provided the counter values are unique and synchronized^52^.

### Hyperchaotic memristive system

The authors of^53^ propose a hyperchaotic memristive circuit that is observed as a non-linear fourth order system of differential equations:

11 $$\begin{aligned} {\left\{ \begin{array}{ll} \dot{x}_1 = a_1x_1 + a_2(x_2 - x_1)x^2_4,\\ \dot{x}_2 = -x_3 - a_3x_2 -a_4(x_2 - x_1)x^2_4,\\ \dot{x}_3 = x_2,\\ \dot{x}_4 = a_5x_4 + a_6(x_2 - x_1) + a_7x_4(x_2 - x_1).\\ \end{array}\right. } \end{aligned}$$

**Fig. 2:**
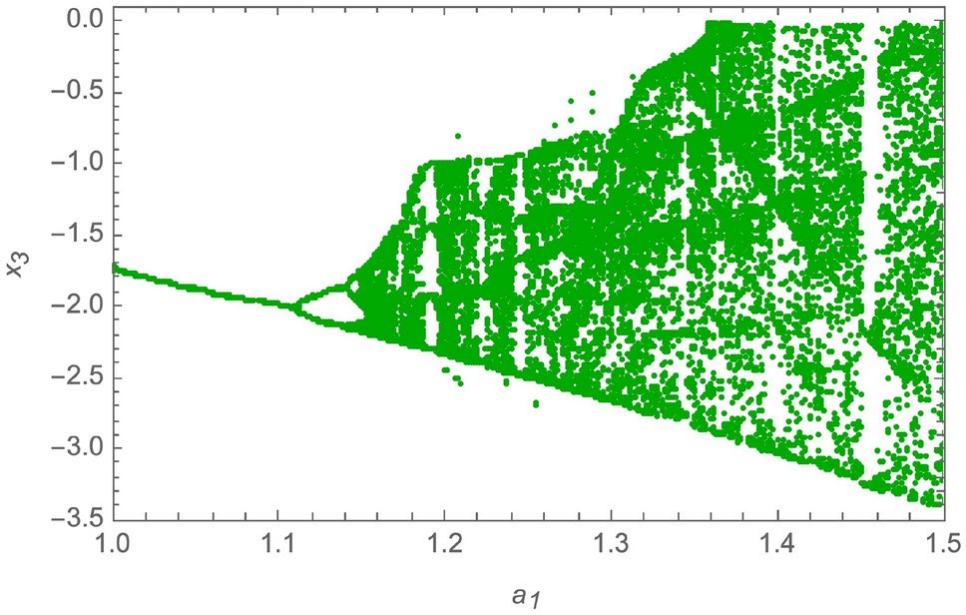
Bifurcation with $$a_1 \in [1, 1.5]$$.

**Fig. 3:**
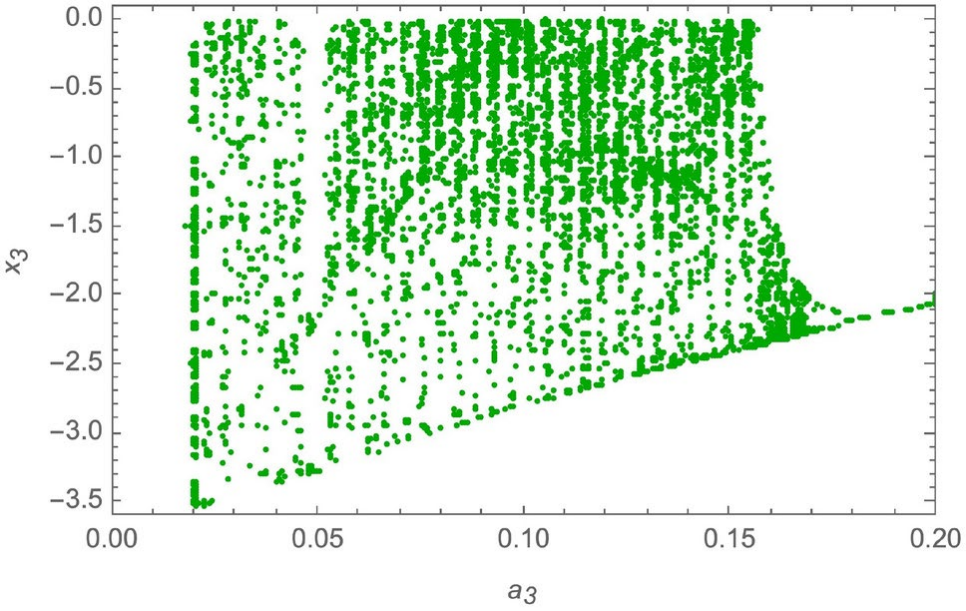
Bifurcation with $$a_3 \in [0.02, 0.2]$$.

**Fig. 4:**
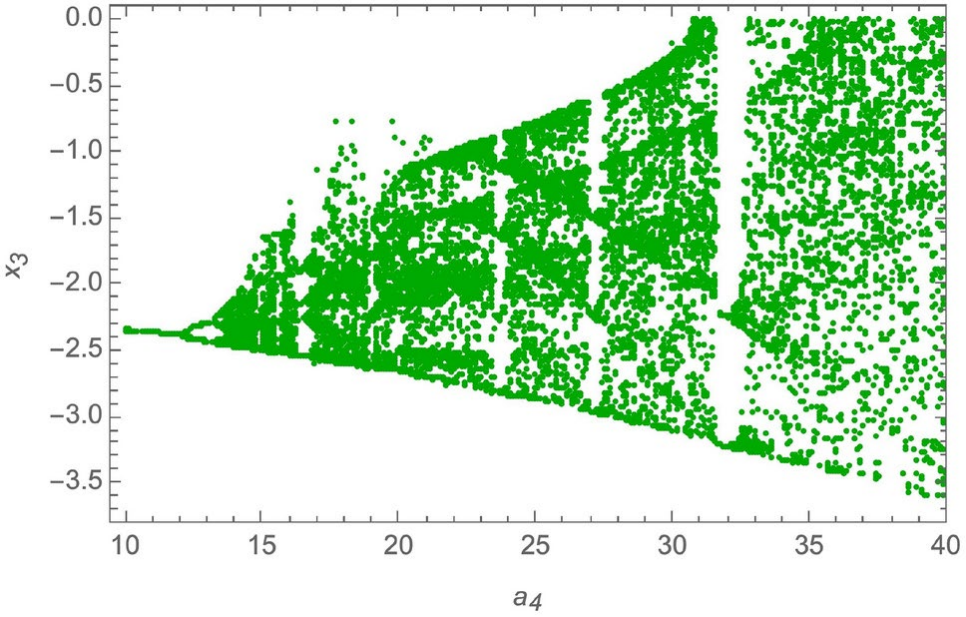
Bifurcation with $$a_4 \in [10, 40]$$.

The parameters of the system in (11) are set to be: $a_1 = 1.5$, $a_2 = 360$, $a_3 = 0.0326$, $a_4 = 36$, $a_5 = -1.5$, $a_6 = -0.0213$, $a_7 = 0.08$. This creates a chaotic attractor that has initial conditions as $x(0) = [0.1, 0.001, 0.05, 0.01]^T$. Also, to determine the dynamic behavior of the non-linear fourth order system and to demonstrate the system’s chaotic behavior, a bifurcation analysis is provided in^53^ as follows:

1. Bifurcation with $a_1 \in [1, 1.55]$, and $a_2 = 360$, $a_3 = 0.0326$, $a_4 = 36$, $a_5 = -1.5$, $a_6 = -0.0213$, $a_7 = 0.08$, is provided in Fig. 2.
2. Bifurcation with $a_3 \in [0.02, 0.2]$, and $a_1 = 1.5$, $a_2 = 360$, $a_4 = 36$, $a_5 = -1.5$, $a_6 = -0.0213$, $a_7 = 0.08$, is provided in Fig. 3.
3. Bifurcation with $a_4 \in [10, 40]$, and $a_1 = 1.5$, $a_2 = 360$, $a_3 = 0.0326$, $a_5 = -1.5$, $a_6 = -0.0213$, $a_7 = 0.08$, is provided in Fig. 4.

**Fig. 5:**
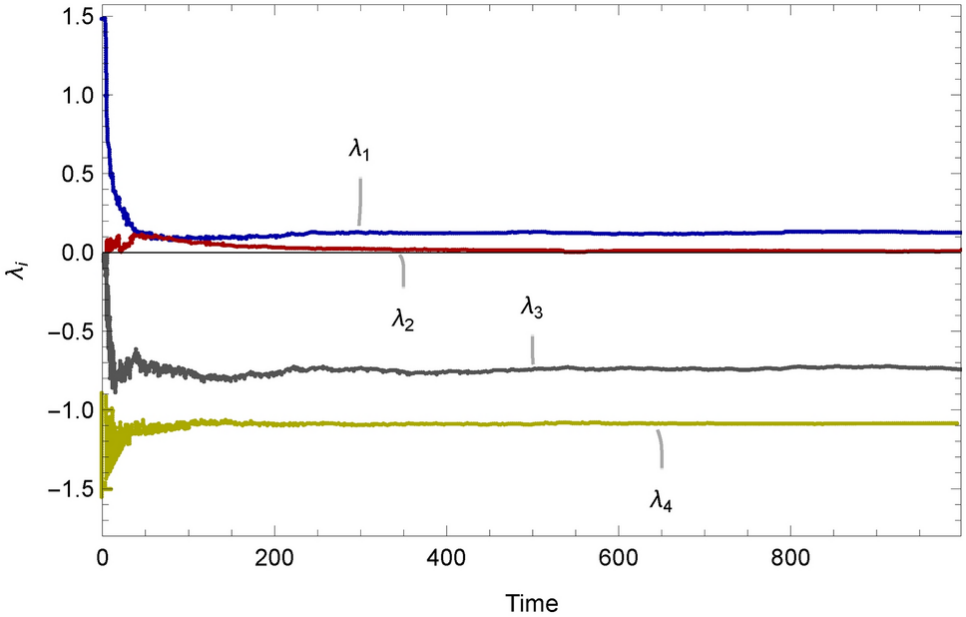
Lyapunov exponents, for $a_1 = 1.5, a_2 = 360, a_3 = 0.0326, a_4 = 36, a_5 = 1.5, a_6 = 0.0213, a_7 = 0.08$.

Furthermore, the authors of^53^ find four Lyapunov exponents, numerically calculated by Wolf’s method to explore the system’s long-time behavior and can be visualized in Fig. 5. These are $L_1 = 0.128162$, $L_2 = 0.013992$, $L_3 = -0.745648$, $L_4 = -1.091846$. The chaotic attractor exhibits two positive Lyapunov exponents along with a fractional Lyapunov dimension, confirming the chaotic behavior of the system in (11).

### Hyperchaotic 6D system

The authors of^54^ propose a six-dimensional hyperchaotic system of differential equations. It can be expressed as:

12 $$\begin{aligned} {\left\{ \begin{array}{ll} \dot{x}_1=h(x_2 -x_1)+x_4,\\ \dot{x}_2=-f x_2 -x_1x_3+x_6,\\ \dot{x}_3=-l+x_1x_2,\\ \dot{x}_4=-x_2 -x_5,\\ \dot{x}_5=kx_2 +x_4,\\ \dot{x}_6=gx_1 +mx_2.\\ \end{array}\right. } \end{aligned}$$

**Fig. 6:**
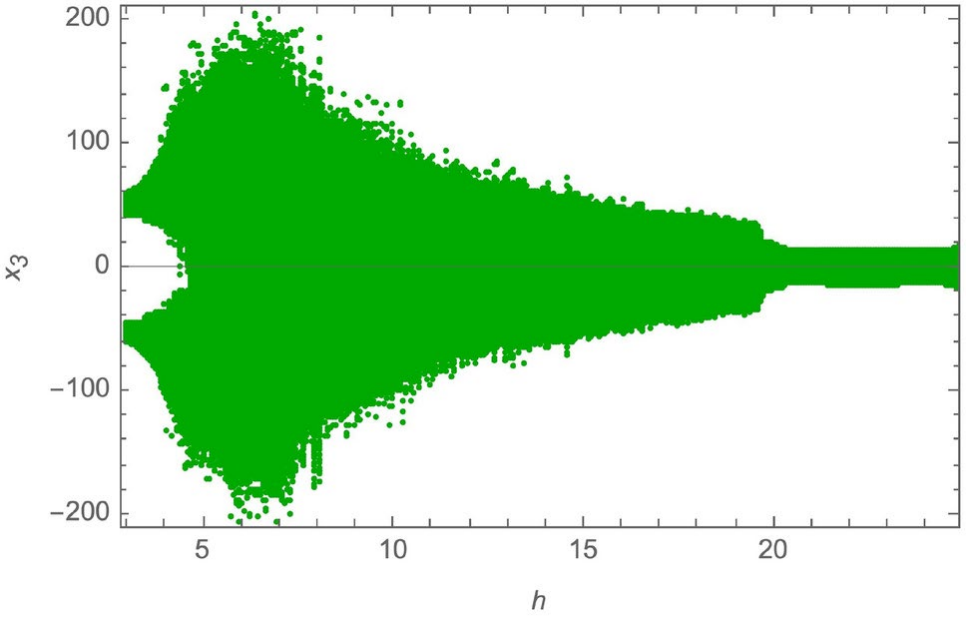
Bifurcation for $l = 100, f = 2.7, k = 2, g = -3, m = 1$, and $h \in [3,25]$.

**Fig. 7:**
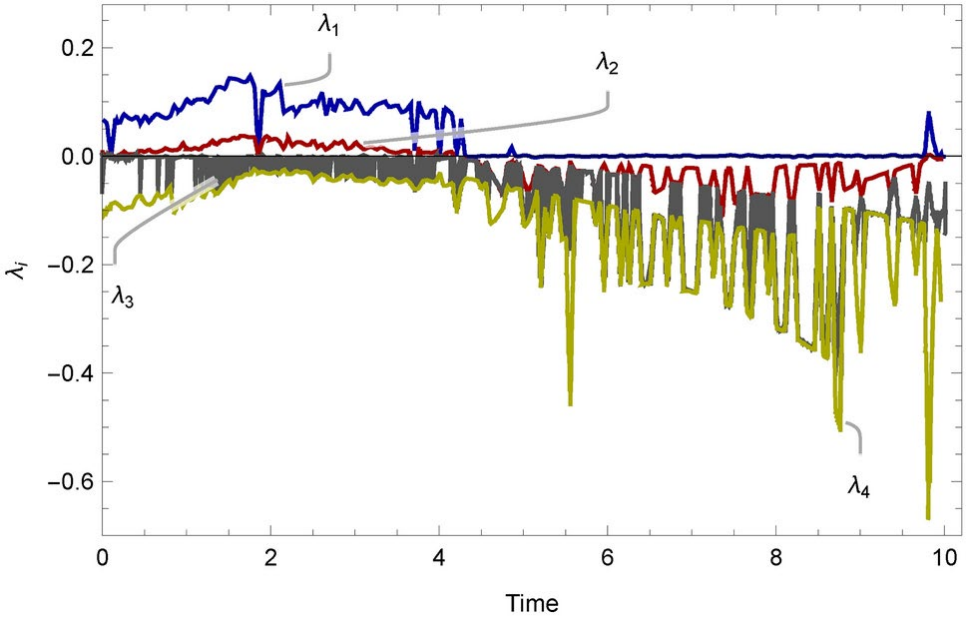
Lyapunov exponents’ plot for $h = 100, l = 80, f = 40, g = -80, m = 72$, and $k \in [0,10]$.

The system variables in (12) are represented by $x_i\ (1\le i\le 6)$ and the system parameters are denoted by $f,g\ne 0,\ h>0,\ k,l>0$ and $m\ne 0$. By selecting the parameters to have the following values $(f,\ g,\ k,\ l,\ m)=(2.7,\ -3,\ 2,\ 100,\ 1)$, the system in (12) provides chaotic behavior, especially if the parameter *h* is within the range of [4.273, 15]. Figure 6 provides the bifurcation diagram for the system in (12). At these certain values, the Lyapunov exponents of the system are $L_1=1.3613,\ L_2=0.0733,\ L_3=0.0478,\ L_4=0.0189,\ L_5=0.0000,\ L_6=-14.2010$. Since there are 4 positive Lyapunov exponents, this system has been proven to exhibit hyperchaotic behavior. Figure 7 displays the Lyapunov exponents’ plot for the system in (12). Further analysis of the dynamics of this system is carried out in^54^, including a verification of randomness of its output via a 0-1 test.

### Hill cipher

The Hill cipher, named after its inventor Lester S. Hill in 1929, represents a significant advancement in the field of classical cryptography. Unlike simpler substitution ciphers that encrypt individual characters, the Hill cipher employs linear algebra to encrypt blocks of text simultaneously, utilizing matrix operations^55^. This method leverages the mathematical structure of matrices, making it one of the earliest examples of polygraphic ciphers in which ciphertext characters depend on multiple plaintext characters. The cipher’s reliance on matrix inversion for decryption introduces a unique blend of cryptographic security and mathematical elegance. Its adaptation to modern cryptographic analysis not only highlights its historical importance but also underscores the enduring relevance of algebraic techniques in image encryption^56^.

For encryption, the Hill cipher translates blocks of plaintext into vectors, which are then multiplied by an encryption matrix *A* modulo *n*, where *n* is the size of the alphabet used. Let $\textbf{P}$ be the plaintext vector and $\textbf{C}$ be the ciphertext vector. The encryption equation is:

13 $$\begin{aligned} \textbf{C} = A \textbf{P} \mod n \end{aligned}$$

Decryption involves the inverse operation. Let $A^{-1}$ be the inverse of the encryption matrix *A*, which is computable if and only if *A* has an inverse modulo *n* (i.e., $\text {gcd}(\text {det}(A), n) = 1$). The decryption equation then is:

14 $$\begin{aligned} \textbf{P} = A^{-1} \textbf{C} \mod n \end{aligned}$$

For both encryption and decryption, the matrices *A* and $A^{-1}$ need to be defined such that their dimensions are compatible with the length of the vector $\textbf{P}$, and *n* typically represents the number of characters in the alphabet (e.g., 26 for the English alphabet). This setup ensures that each letter (or block of letters, depending on the size of the matrix) from the plaintext is systematically transformed into the ciphertext, leveraging matrix algebra for cryptographic processes.

### Blum blum shub

The BBS PRNG, devised by Lenore Blum, Manuel Blum, and Michael Shub in 1986, is renowned for its robust security profile in cryptographic applications^57^. It is predicated on the computational hardness of the integer factoring problem, making it particularly adept at generating high-quality pseudo-random numbers suitable for cryptographic uses. The BBS algorithm operates by repeatedly squaring a number modulo a large composite *N*, where $N = pq$ and *p* and *q* are large, distinct primes chosen such that $p \equiv 3 \pmod {4}$ and $q \equiv 3 \pmod {4}$. These primes are known as Blum primes. The sequence generation formula is given by:

15 $$\begin{aligned} x_{n+1} = x_n^2 \mod N \end{aligned}$$

where $x_0$ is the seed, which must be a coprime relative to *N*. The output of the PRNG is typically derived from the least significant bits of each $x_n$. The security of the output relies significantly on the choice of *N* and the size of the primes involved. The core computational assumption behind BBS is based on the quadratic residuosity problem, asserting that determining whether a number is a quadratic residue modulo *N*, without knowing the factorization of *N*, is computationally infeasible. This foundational principle renders the BBS PRNG particularly resistant to reverse engineering and cryptographic attacks, establishing it as one of the most secure PRNGs when configured with appropriately large prime numbers^58^.

## Proposed MIE algorithm

This section outlines the framework of the proposed MIE algorithm, detailing the steps for creating augmented satellite images and the processes for encrypting and decrypting these images. Each component is detailed in a separate subsection, specifically Section “Augmented satellite image preprocessing” for augmented satellite image preprocessing, Section “Encryption process” for the encryption process, and Section “Decryption process” for the decryption process.

### Augmented satellite image preprocessing

**Fig. 8:**
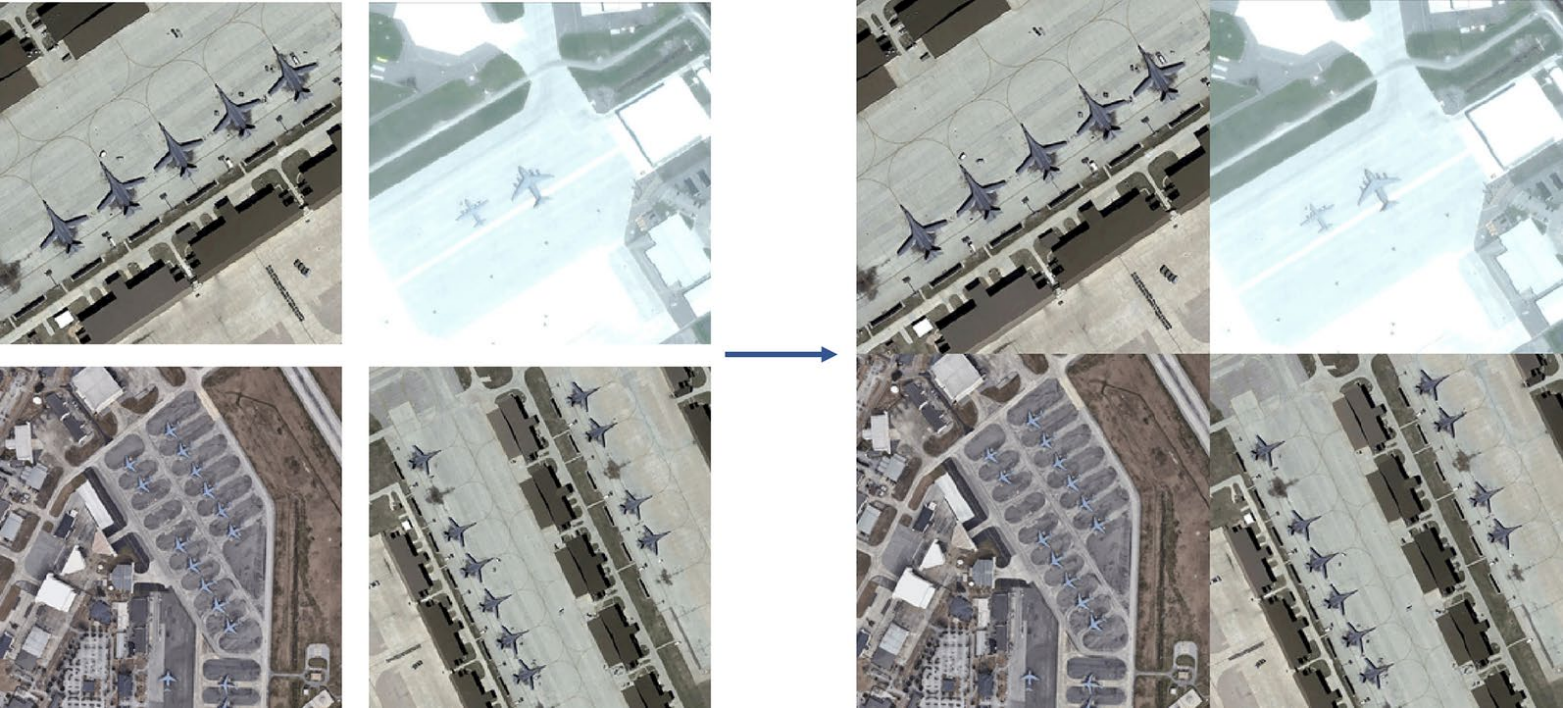
Augmenting multiple satellite images into one (MAR 20 dataset: https://gcheng-nwpu.github.io/).

The MIE algorithm begins with a preprocessing phase that accepts several satellite images as input and produces a single enhanced image as output. Figure 8 visually depicts this process, showing an example where 4 input images, each of size $256\times 256$, are combined to form one augmented output image with dimensions of $512\times 512$. The number of images in each row or column in the augmented image is denoted by *K*. In Fig. 8, $K=2$.

### Counter mode based RC5

The proposed enhancement to the RC5 encryption algorithm integrates a counter mode and employs dynamic rotations based on a chaotic sequence to robustly encrypt image data. The process begins by generating a 128-bit random number as the initial counter, which is encrypted using the RC5 algorithm over several rounds to produce a 128-bit output. The process is repeated for each succeeding counter, which is incremented subsequent to each cryptographic cycle. The total number of counters required is calculated based on the image dimensions, specifically $N \times M \times 8 / 128$, ensuring adequate pseudo-random bits are generated to cover the entire image. Concurrently, a chaotic sequence is generated and scaled between 1 and 6, influencing the amount and direction of rotations applied during the encryption. Each value from this sequence, denoted as $k$, directs the RC5 function by selecting the LSBs from each half of the counter, converting these bits into an integer that determines the rotation applied to the counterpart half. The direction of rotation is contingent on whether $k$ is odd (rotating left) or even (rotating right). Ultimately, the pseudo-random bits obtained from this sophisticated encryption process are used to XOR with the image bits, resulting in a securely encrypted image. This modification not only enhances the traditional RC5 by introducing a counter-based approach but also leverages the unpredictability of chaotic dynamics to adjust the encryption mechanism dynamically, significantly bolstering security. Moreover, this approach accelerates the encryption process as the entire sequence of counters is generated once when the system initiates, allowing for rapid sequential processing without the need for regenerating keys for each block of data. The use of the counter mode offers additional advantages, including the ability to preprocess counters and parallelize operations, further enhancing throughput and efficiency in scenarios requiring high volumes of data encryption, such as in image processing applications^59^.

### Chaos based blum blum shub

The modified algorithm for generating cryptographic parameters $p$, $q$, and $x_0$ for the BBS PRNG utilizes a chaotic sequence to enhance randomness and security. Initially, a 1536-bit chaotic sequence is generated and split into three 512-bit segments. The first two segments are adjusted to produce the primes $p$ and $q$, each of which must satisfy the condition $p, q \equiv 3 \pmod {4}$. This is achieved by first converting each 512-bit segment into an integer, adjusting it to ensure it is congruent to 3 modulo 4, and then using a prime-search loop that increments by 4 to preserve the congruence while seeking the next prime number. The last segment is used to generate $x_0$, which is also set to be a prime to ensure it is coprime with $n = pq$. This seed $x_0$ is then used in the BBS algorithm to generate a pseudo-random sequence, with each bit of the sequence dynamically selected based on a secondary chaotic sequence, scaled to select from the full bit range of $x_{i+1}$ in the BBS outputs. This process adds a layer of complexity and randomness to the encryption progression, augmenting security by leveraging the characteristic capriciousness as well as sensitivity to initial conditions of chaotic systems.

### Chaos based Hill cipher

The implementation of a chaotic-based Hill cipher for image encryption innovatively combines chaos theory with classical cryptographic methods. This approach entails dividing the target image into manageable blocks, each of which will be encrypted separately. The encryption process for each block uses a unique matrix, generated from a chaotic sequence, which ensures robust security through high entropy and unpredictability inherent in chaotic systems.

To guarantee the functionality and security of the encryption process, it is crucial that each matrix used is invertible modulo 256. The number 256, being a power of two, presents unique characteristics, notably that its only odd factor is 1. This property simplifies the invertibility condition of the encryption matrices: a matrix with an odd determinant will always have a greatest common divisor of 1 with 256 ($\text {gcd}(\text {det}(A), 256) = 1$). Consequently, ensuring the matrix has an odd determinant guarantees its invertibility under modulo 256 operations, a critical aspect for the decryption phase of the Hill cipher.

The encryption matrix for each block is derived from a chaotic sequence. This sequence is first generated to a considerable length, depending on the total number of image blocks to be encrypted. Each value in the chaotic sequence is then scaled to fit within the 0 to 255 range, suitable for operations under modulo 256. These scaled values are segmented into sublists, each consisting of four elements. The specific requirement for each sublist is to follow an odd, even, even, odd pattern. This pattern is pivotal as it ensures that when the elements are arranged into a $2\times 2$ matrix, the matrix will inherently possess an odd determinant. The odd determinant is key to satisfying the invertibility condition necessary for both encryption and subsequent decryption processes.

### Preprocessing for noise removal

The following subsections describe the noise characteristics in satellite imagery, as well as possible means to reduce such noise, effectively and efficiently, as a preprocessing step prior to encryption.

#### Noise characteristics in satellite imaging

In satellite imagery, noise is predominantly introduced during the digital conversion process, where optical images are transformed into electrical signals and subsequently digitized. The nature of noise can vary significantly depending on the specifics of the image acquisition and processing chain, but typical manifestations in satellite images include:

- Gaussian Noise: Randomly distributed, this noise affects the image as white noise variations due to its normal distribution properties^60^.
- Salt & Pepper Noise: Arises from sharp and sudden disruptions in the image signal, typically due to errors in the digitization process or sensor faults, manifesting as sparsely occurring white and black pixels^60^.
- Speckle Noise: Common in radar and synthetic aperture radar (SAR) imagery, speckle is caused by the coherent processing of backscattered signals and can severely impact the image quality^61^.

#### Advanced filtering techniques

Effective noise reduction in satellite imagery requires sophisticated filtering techniques that can adapt to the varying characteristics of noise across different sensors and conditions^62,63^:

- Gaussian Filter: Utilized for its efficacy in blurring and noise reduction through a weighted average, where the weights are determined by a Gaussian function, optimizing the balance between noise smoothing and edge preservation.
- Mean Filter: Although basic, it provides a quick and effective means of reducing high-frequency noise by averaging pixel values within a defined kernel.
- Median Filter: Particularly useful for non-Gaussian noise types like salt & pepper, this non-linear filter replaces each pixel value with the median of neighboring pixel values, preserving edges while reducing noise.
- Adaptive Median Filter: Enhances the median filtering approach by dynamically adjusting the kernel size based on local variance, allowing for more effective noise reduction in heterogeneous noise environments.
- Adaptive Wiener Filter: Tailors filtering parameters to the local image variance, optimizing noise reduction based on the statistical characteristics of each pixel neighborhood. This adaptive approach is superior for handling complex noise models and maintaining image details.
- Mittag-Leffler 2D Filter: This advanced filter leverages the Mittag-Leffler function, a generalization of exponential functions, to address issues in fractional-order systems and processes. In image processing, it is particularly effective for handling images with anomalous diffusion characteristics, often encountered in complex dynamic environments. The filter’s ability to adapt to fractional noise models makes it ideal for enhancing image clarity in scenarios where traditional integer-order filters fall short. This approach provides superior performance in preserving image details while effectively reducing correlated and non-Gaussian noise types.

For satellite imagery analysis, selecting and tuning these filters according to specific noise characteristics and desired image quality outcomes is crucial^64^. This not only enhances the visual quality but also improves the reliability of subsequent image processing tasks such as encryption, feature extraction and classification.

### Encryption process

The following steps describe the encryption process.

1. *Input Image*: Denote the augmented plain image as $I$.
2. *Channel Separation*: Decompose $I$ into three primary color channels:
  - $I_R$ for the Red channel,
  - $I_G$ for the Green channel,
  - $I_B$ for the Blue channel.
3. For each channel $I_c$ (where $c \in \{R, G, B\}$):
  - *Chaotic Sequence Generation*: Generate a chaotic sequence from the memristor system, denoted by $CS_c$.
  - *SVD Transformation*: Apply SVD Transformation as described in Algorithm 1 to $CS_c$ to derive a key matrix, denoted by $K_c$.
  - *Modular Additive Confusion*: Apply modular additive confusion to $I_c$ using $K_c$, resulting in confused image data $D_c$.
  - *Convert Image to Bitstream*: Convert the diffused image data $D_c$ into a continuous bitstream, denoted by $B_c$.
  - *Generate Another Chaotic Sequence*: Produce a new sequence from the memristor system, denoted by $CS'_c$.
  - *Generate Initial Counter*: Create a 128-bit random number as the initial counter, denoted by $C_{ic}$.
  - *RC5 Encryption with Counter Mode*: Use $CS'_c$ and $C_{ic}$ to operate a counter mode-based RC5 encryption, as discussed in section “Counter mode based RC5”, producing a cryptographic bitstream $X_c$.
  - *XOR Operation*: Combine $X_c$ with $B_c$, resulting in an XOR’d bitstream $B'_c$.
  - *Generate Hyperchaotic Sequence*: Produce a sequence using a 6D hyperchaotic system, denoted by $CS''_c.$
  - *Matrix Formation*: Use $CS''_c$ to form a list of $2 \times 2$ matrices $M_c$ as described in Algorithm 2, ensuring each matrix is invertible.
  - *Convert Bit-stream to Image*: Convert $B'_c$ back into image format.
  - *Image Segmentation*: Reshape the converted image data into $2 \times 2$ blocks.
  - *Hill Cipher Encryption*: Apply the Hill cipher to each $2 \times 2$ block using matrices $M_c$, resulting in encrypted image blocks $E_c$.
  - *Generate Two Hyperchaotic Sequences*: Create two sequences from the 6D hyperchaotic system; one for modified BBS parameters $CS'''_c$, and one as a selector $Sel_c$.
  - *Modified BBS Operation*: Use $CS'''_c$ and $Sel_c$ to run the process as described in Algorithm 3, and Algorithm 4, generating $S_c$.
  - *S-box Generation*: Form an S-box from $S_c$ using Algorithm 5. Table 2, 3 and 4 are examples of three generated S-boxes for the three channels.
  - *S-box Application*: Apply the previously generated S-box to $E_c$, resulting in the final encrypted channel $F_c$.
4. *Recombine Channels*: Combine the encrypted channels $F_R$, $F_G$, and $F_B$ to form the final encrypted image $F$.

**Fig. 9:**
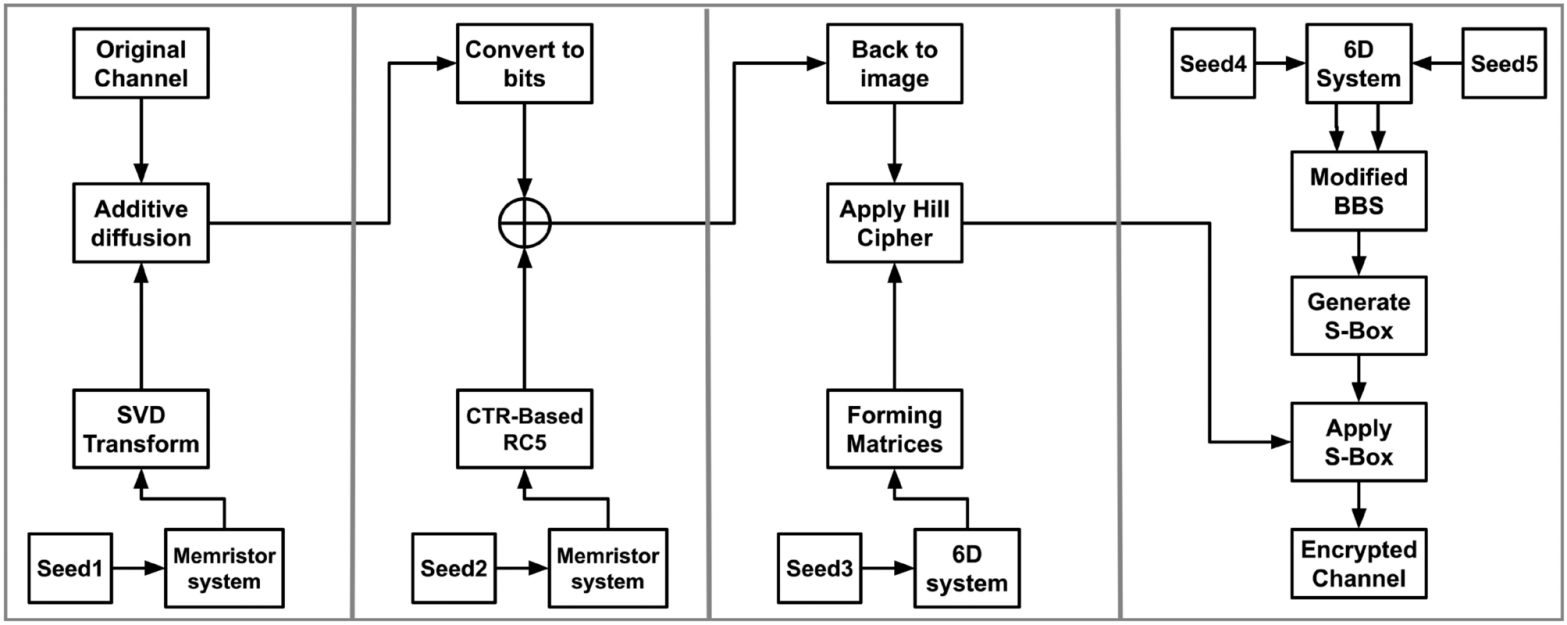
Encryption process.

Figure 9 visually illustrates the encryption process.

### Decryption process

The following steps describe the decryption process.

1. *Input Image*: Denote the augmented encrypted image as $F$.
2. *Channel Separation*: Decompose $F$ into three primary color channels:
  - $F_R$ for the Red channel,
  - $F_G$ for the Green channel,
  - $F_B$ for the Blue channel.
3. *For each channel *$F_c$ (*where* $c \in \{R, G, B\}$):
  - *Generate Two Hyperchaotic Sequences*: Create two sequences from the 6D hyperchaotic system; one for modified BBS parameters $CS'''_c$, and one as a selector $Sel_c$.
  - *Modified BBS Operation*: Use $CS'''_c$ and $Sel_c$ to run the process as described in Algorithm 3, and Algorithm 4, generating $S_c$, generating $S_c$.
  - *S-box Generation*: Form an S-box from $S_c$ using Algorithm 5.
  - *S-box Application*: Apply the inverse of the previously generated S-box to $F_c$, resulting in $E_c$.
  - *Generate Hyperchaotic Sequence*: Produce a sequence using a 6D hyperchaotic system, denoted by $CS''_c$.
  - *Matrix Formation*: Use $CS''_c$ to form a list of $2 \times 2$ matrices $M_c$ as described in Algorithm 2, ensuring each matrix is invertible.
  - *Convert Bitstream to Image*: Convert $X_c$ back into image format.
  - *Image Segmentation*: Reshape the converted image data into $2 \times 2$ blocks.
  - *Hill Cipher Decryption*: Apply the Hill cipher to each $2 \times 2$ block using the inverse of each matrix in $M_c.$
  - *Convert image into bit stream*: convert the image into a bit-stream, resulting in $B'_c$.
  - *Generate a Chaotic Sequence*: Produce a new sequence from the memristor system, denoted by $CS'_c$.
  - *Generate Initial Counter*: Create a 128-bit random number as the initial counter, denoted by $C_{ic}$.
  - *RC5 Encryption with Counter Mode*: Use $CS'_c$ and $C_{ic}$ to operate a counter mode-based RC5 encryption as described in Section “Counter mode based RC5”, producing a cryptographic bitstream $X_c$.
  - *XOR Operation*: Combine $X_c$ with $B'_c$, resulting in an XOR’d bitstream $B_c$.
  - *Convert bitsream to image*: Convert $B_c$ to an image, resulting in $D_c$.
  - *Chaotic Sequence Generation*: Generate a chaotic sequence from the memristor system, denoted by $CS_c$.
  - *SVD Transformation*: Apply SVD to $CS_c$ to derive a key matrix using Algorithm 1, denoted by $K_c$.
  - *Modular Subtractive Diffusion*: Apply modular subtractive diffusion to $D_c$ using $K_c$, resulting in diffused image data $I_c$.
4. *Recombine Channels*: Combine the encrypted channels $I_R$, $I_G$, and $I_B$ to form the final encrypted image $I$.

**Figure 10:**
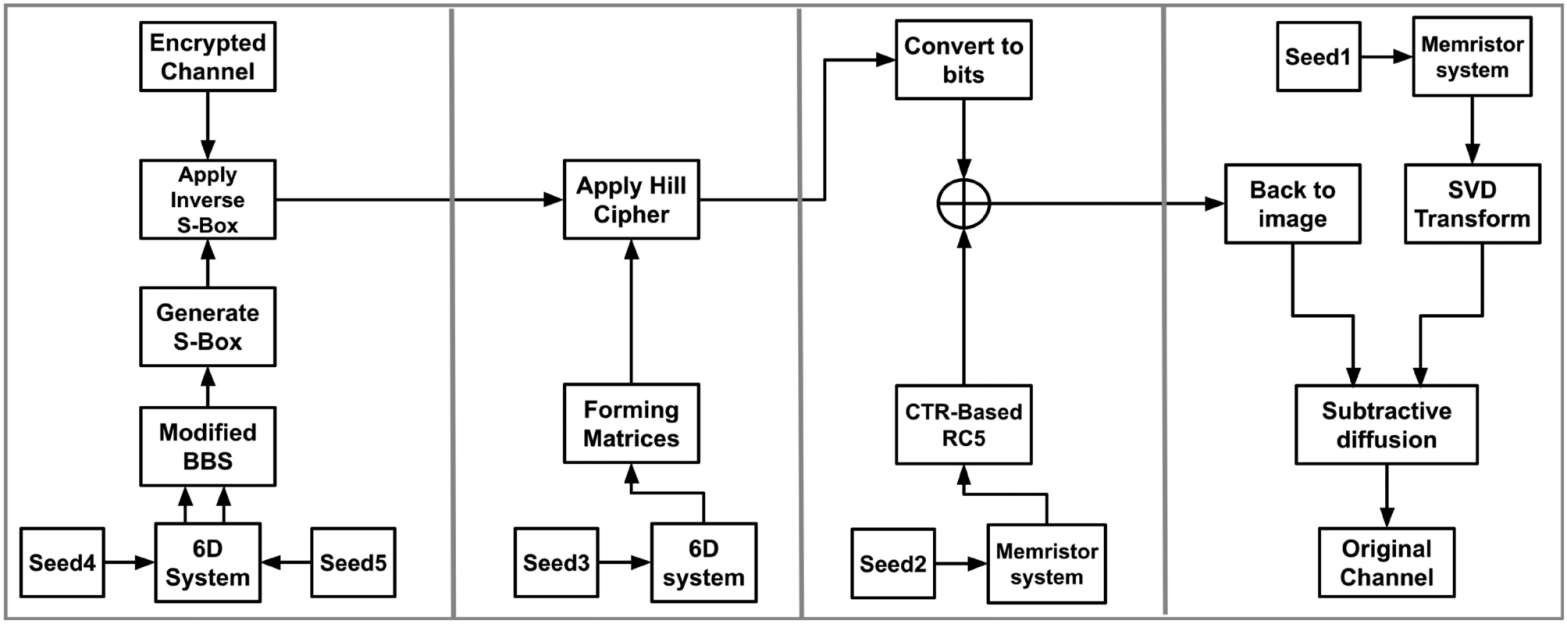
Decryption process.

Figure 10 visually illustrates the decryption process.

**Algorithm 1:**
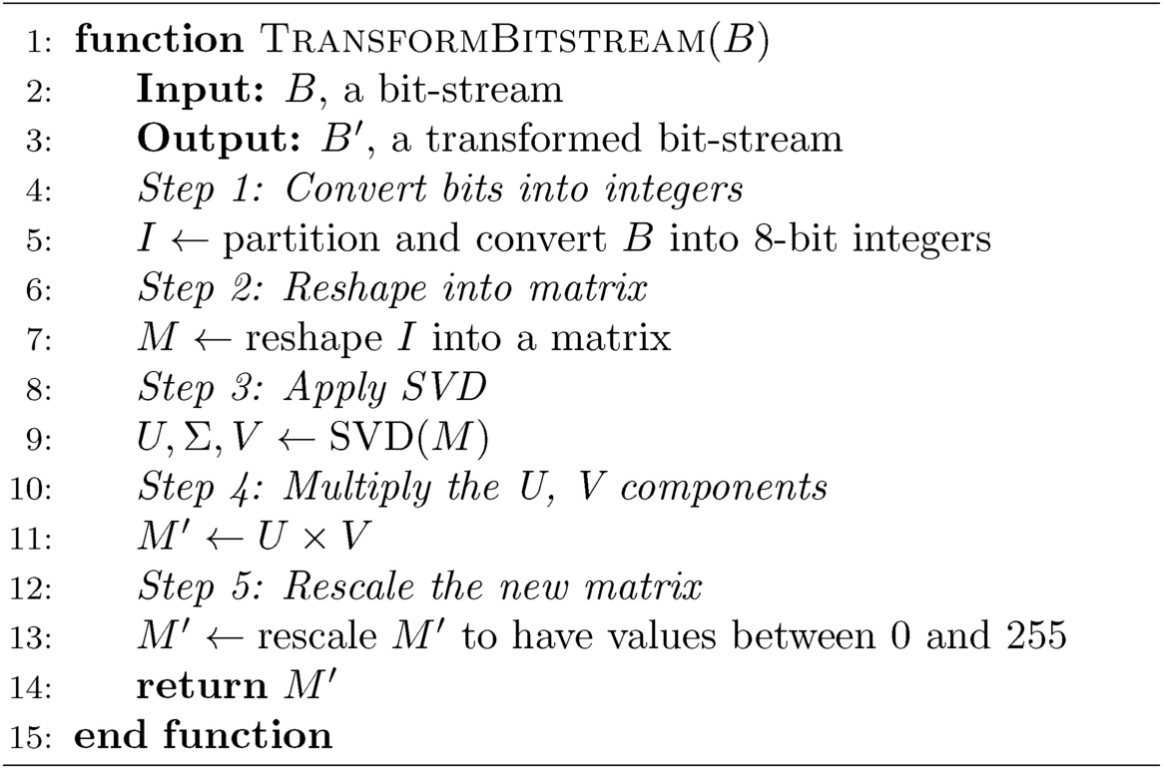
Bit-stream transformation function

**Algorithm 2:**
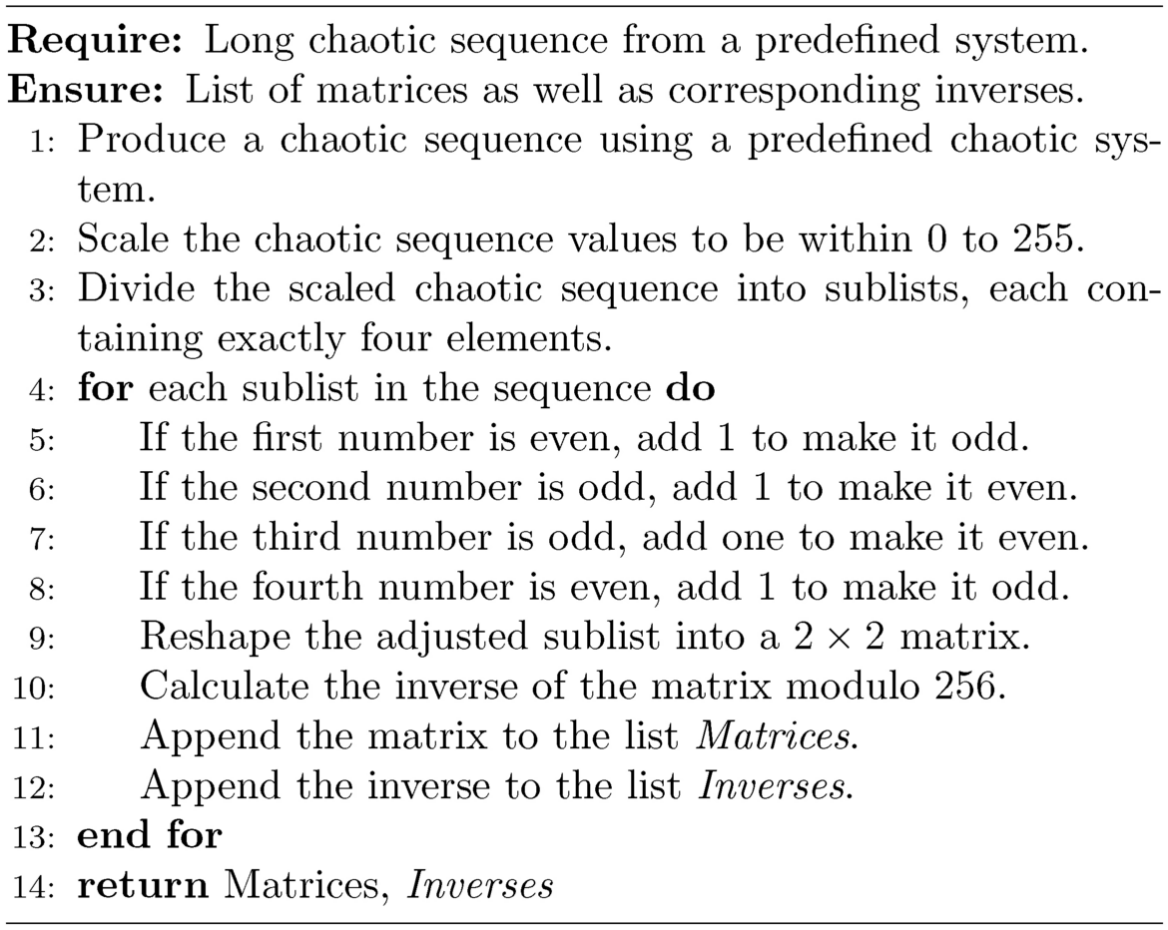
Chaotic-based Hill cipher

**Algorithm 3:**
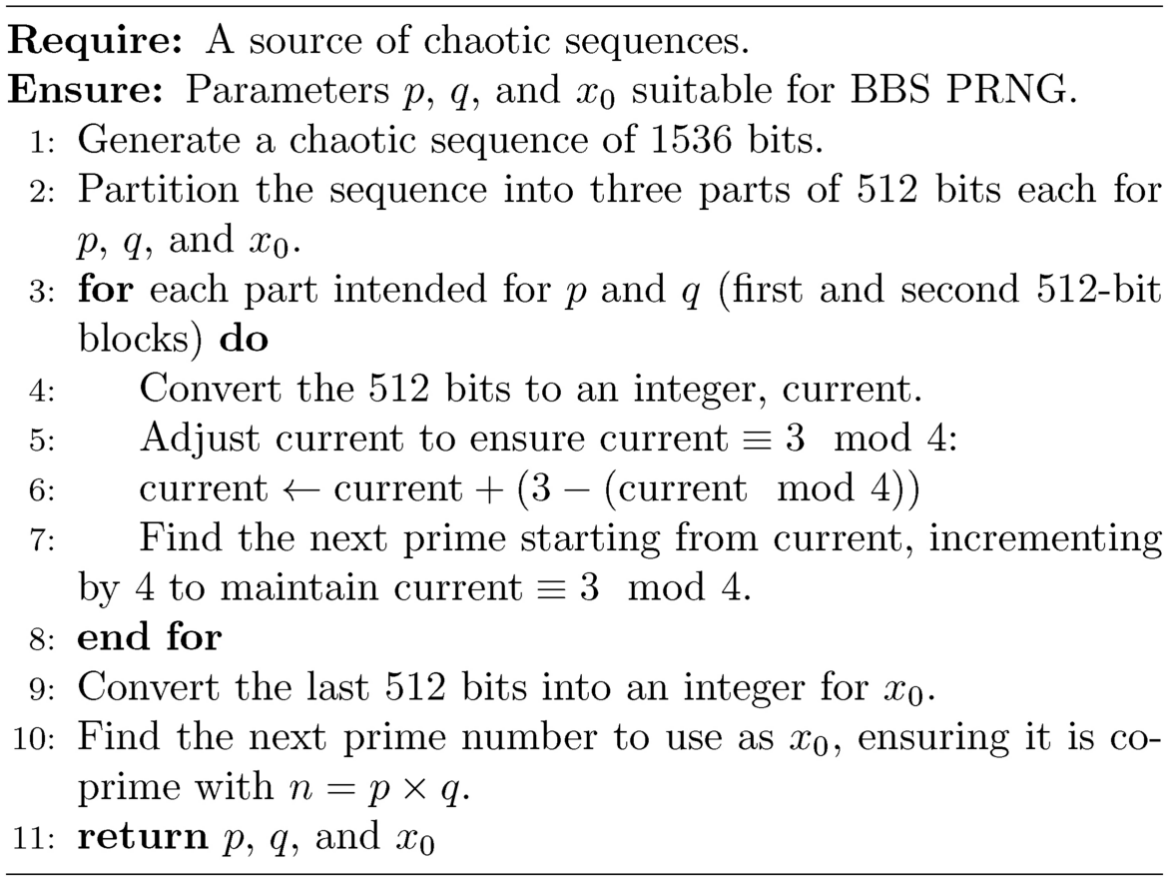
Generate cryptographic parameters $p$, $q$, and $x_0$ for BBS PRNG

**Algorithm 4:**
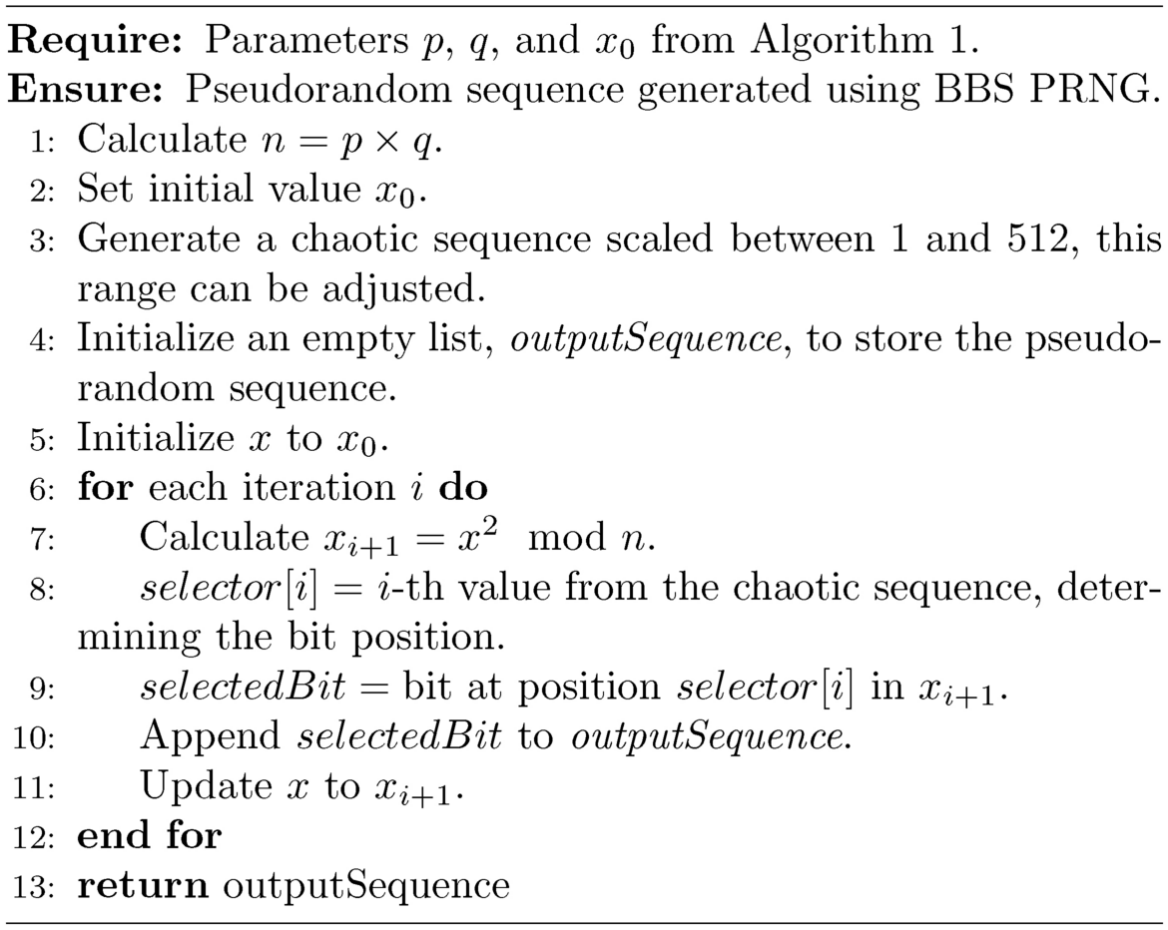
Generate BBS pseudorandom sequence with dynamic bit selection

**Algorithm 5:**
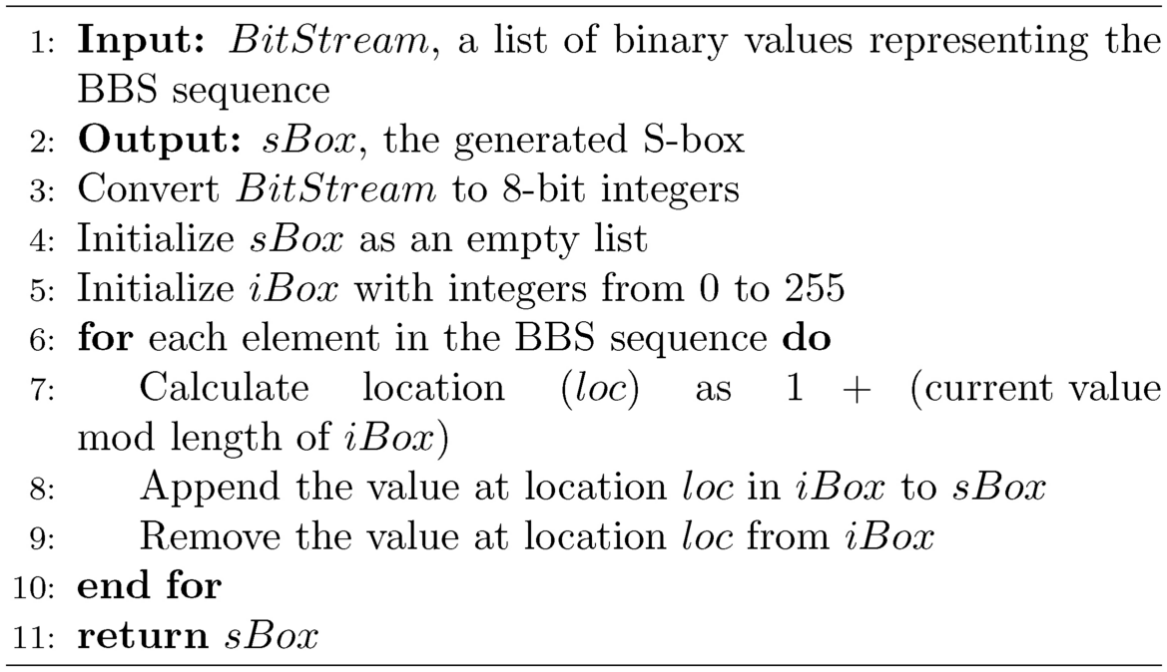
Construct an S-box given a bit-stream *BitStream*. Adopted from^26^

**Table 2:** The first proposed S-box constructed from a sequence generated by modified BBS.

93	249	190	56	81	75	208	37	99	184	59	62	148	153	70	16
24	4	94	164	223	119	243	209	222	67	238	224	84	235	242	31
241	47	226	33	108	250	193	227	15	230	189	107	7	239	98	100
45	57	77	131	143	174	12	161	180	52	205	39	96	22	76	120
251	158	1	225	54	215	110	91	85	10	160	201	2	63	121	151
72	244	60	43	49	124	126	20	5	117	139	210	103	212	219	83
55	28	40	168	115	69	112	38	105	191	26	182	104	203	111	87
109	236	142	144	154	32	149	73	163	147	11	137	66	138	125	204
128	14	240	169	175	247	113	130	196	0	48	118	167	129	13	217
159	176	206	88	228	231	79	140	172	90	177	132	51	30	178	192
211	237	78	232	213	9	166	19	97	199	229	6	220	245	214	116
46	221	186	86	17	102	150	254	74	253	165	95	162	92	114	207
234	157	8	122	171	133	35	155	156	152	200	188	41	27	23	80
218	170	42	197	181	3	29	123	145	183	58	71	141	195	136	106
179	61	68	185	50	198	82	64	233	134	135	187	255	246	53	173
101	252	44	65	202	127	146	248	36	34	216	25	21	89	194	18

**Table 3:** The second proposed S-box constructed from a sequence generated by modified BBS.

113	250	190	127	4	14	122	1	77	3	153	151	143	15	69	6
171	205	222	61	12	147	53	189	34	24	95	63	226	26	111	75
36	17	243	218	191	16	238	19	31	199	91	193	8	115	43	123
185	73	49	104	177	29	74	200	163	9	67	212	179	251	168	10
33	45	206	217	233	82	83	39	86	76	124	246	88	99	175	112
18	219	71	78	156	93	221	58	46	145	5	32	152	198	103	183
144	176	211	13	28	146	65	120	125	182	96	50	121	137	129	180
109	157	55	40	38	162	85	136	184	150	235	48	118	22	154	229
2	30	133	187	128	102	196	234	131	178	119	47	23	164	41	57
68	79	35	228	134	216	245	188	92	7	101	158	138	159	225	220
139	170	81	126	237	84	207	236	202	213	223	42	242	114	141	98
161	230	210	62	248	97	51	169	240	52	194	166	255	165	107	173
241	239	149	80	72	87	100	105	186	201	110	59	231	25	197	249
195	116	132	172	60	252	106	181	70	209	142	140	203	155	130	27
54	224	160	90	11	20	89	56	192	37	247	204	117	148	66	254
21	167	253	108	227	44	64	215	214	208	0	135	174	244	94	232

**Table 4:** The third proposed S-box constructed from a sequence generated by modified BBS.

45	246	35	66	106	168	222	199	100	159	121	242	173	4	128	213
214	244	85	86	223	206	151	11	25	87	51	143	180	91	89	237
167	7	248	28	71	52	227	164	194	56	148	233	75	18	208	129
92	252	224	161	72	251	198	5	67	169	32	82	124	63	78	172
24	50	131	14	27	103	118	69	105	93	30	8	23	170	211	97
12	10	115	122	94	189	76	81	40	41	95	140	142	113	49	1
219	70	136	79	209	147	107	141	68	84	54	73	26	120	225	185
183	155	197	195	22	37	55	175	48	218	255	192	111	240	88	184
239	60	110	102	191	44	109	98	108	253	47	36	114	144	220	20
125	2	123	19	133	226	221	134	149	201	83	146	61	215	62	64
152	187	42	212	101	230	57	245	205	137	176	17	181	59	166	207
46	104	202	186	58	243	178	196	138	127	182	139	3	126	135	254
165	132	39	38	31	116	117	53	160	163	203	190	193	16	0	119
158	9	156	217	6	210	229	90	238	154	150	34	234	99	157	77
235	153	171	29	162	21	247	250	216	130	228	65	188	43	13	174
232	179	96	249	231	241	204	145	74	33	15	177	112	200	236	80

## Performance evaluation and numerical results

In this section, a comprehensive analysis is performed using a computer equipped with an Intel^®^ Core™ *i*7-7500U CPU operating at 2.7 GHz and equipped with 8 GB of RAM. Unless otherwise noted, the processed images are augmented and resized to dimensions of $256 \times 256$ pixels with $K=4$. These images were obtained from two online repositories: the USC-SIPI database^65^ and the MAR20 database^66^.

Metrics like Mean Squared Error (MSE), Peak Signal-to-Noise Ratio (PSNR), and Mean Absolute Error (MAE) are used to gauge image distortion and pixel discrepancies. The randomness and resistance to statistical attacks of the encrypted images are measured through entropy tests. Furthermore, DFT analysis is performed to identify patterns in the frequency domain, and pixel cross-correlation tests are utilized to assess the disruption of spatial relationships within the images. Additionally, the proposed MIE algorithm’s sensitivity to input changes, the extensive key space to thwart brute-force attacks, and NIST statistical tests are applied to evaluate the randomness of the encrypted images.

The effectiveness of the proposed MIE algorithm is evaluated based on encryption time, and the performance of the S-box is analyzed for NL and complexity, among other factors. For these analyses, Wolfram Mathematica^®^ version 13.1, which is recognized for its parallel processing abilities, is employed. Collectively, these evaluations provide a thorough assessment of the proposed MIE algorithm, highlighting its proficiency and effectiveness in protecting satellite images.

### Visual analysis

**Fig. 11:**
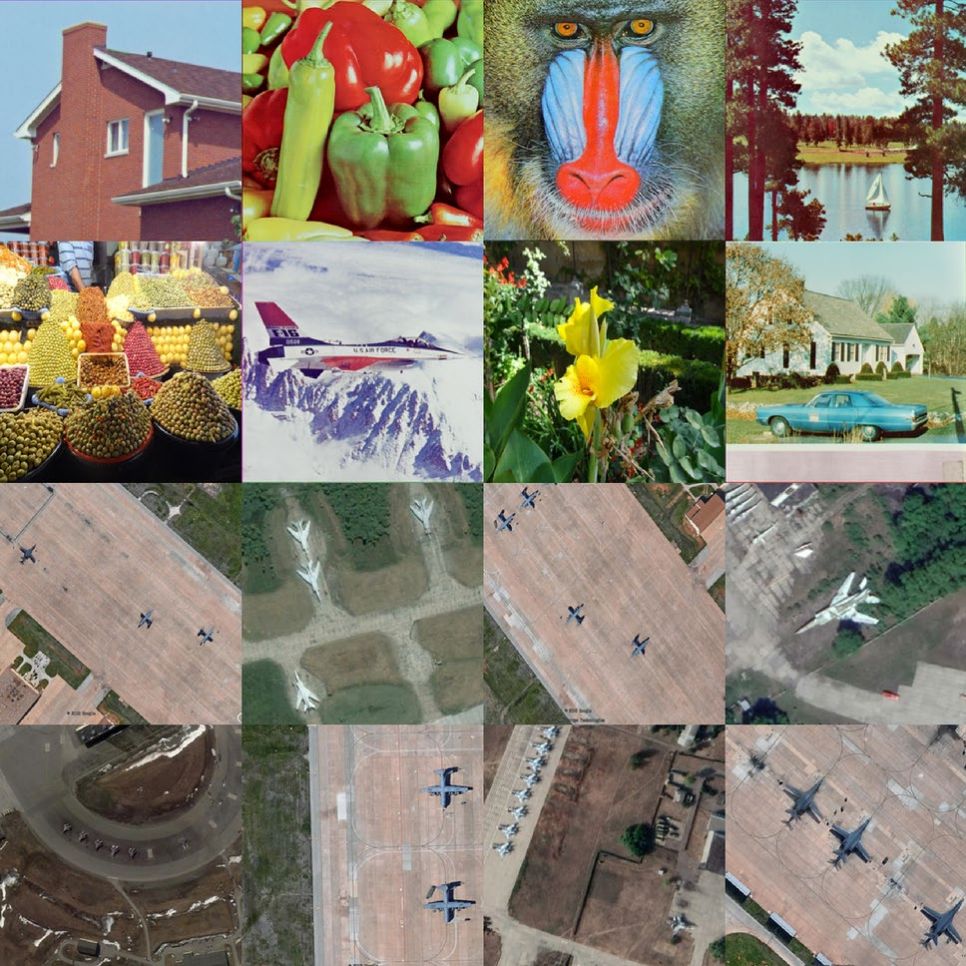
Plain augmented image formed for $K=4$ (16 plain images, obtained from the USC-SIPI image database: https://sipi.usc.edu/database/ and the MAR 20 dataset: https://gcheng-nwpu.github.io/).

**Fig. 12:**
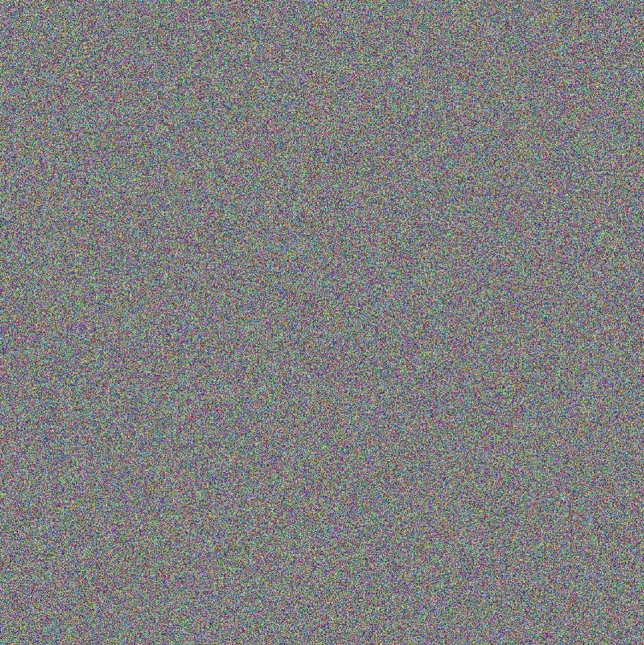
Encrypted augmented image formed for $K=4$ (16 encrypted images).

In this analysis, the focus is on how an augmented image is transformed by comparing its original and encrypted versions, as depicted in Figs. 11 and 12 respectively. The original image shown in Fig. 11 consists of an augmentation of 16 images, each characterized by rich details, sharpness, and contrast. However, once encrypted as displayed in Fig. 12, these distinct features are masked, converting the image into what appears to be a random assortment of pixel values. This transformation highlights the proposed MIE algorithm’s efficacy in effectively obscuring visual information to secure that the original content remains concealed.

### Histogram analysis

Histogram analysis plays a vital role in assessing the strength and robustness of the proposed MIE algorithm. Histograms offer a visual representation of the distribution of pixel intensities within an image. For an encryption procedure to be operative and secure, the encrypted image histogram should show a uniform distribution. This uniformity is crucial as it conceals any statistical clues that could be exploited for cryptanalysis.

**Fig. 13:**
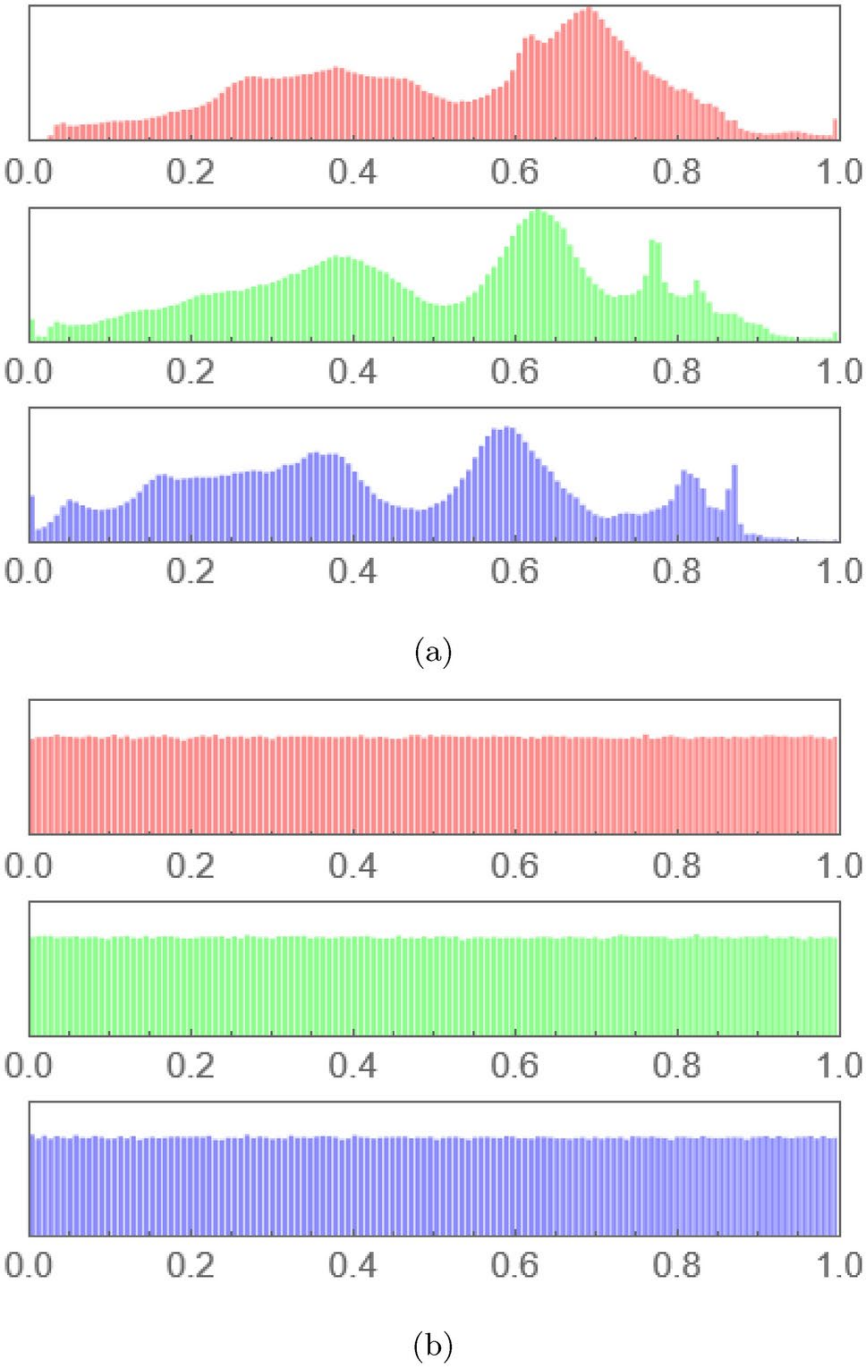
Comparison of histogram plots of augmented images: (**a**) plain and (**b**) encrypted.

Figure 13a presents the histogram of the plain augmented image, which illustrates the typical peaks and troughs associated with the frequency of each pixel intensity. In contrast, Fig. 13b depicts the histogram of the encrypted version of the same image. The uniformity and flatness observed in this histogram reflect the effectiveness of the proposed MIE algorithm, showing that the pixel intensities are evenly distributed and that any discernible patterns from the original image are successfully obscured. The pictorial suggestion evidenced by these histograms powerfully confirms the declaration that the cryptographic technique proposed efficiently randomizes pixel values. This randomization significantly enhances the security of the encrypted images, making them more resilient against statistical attacks.

### Mean squared error

In fact, the MSE is a metric that quantifies the average squared difference between the pixels of the original image and the encrypted image. This numerical value serves as an indicator of the distortion or change introduced by the encryption process. The formula for calculating the MSE is commonly expressed as:

16 $$\begin{aligned} MSE=\frac{1}{M\times N}\sum _{i=0}^{M-1}\sum _{j=0}^{N-1}(P_{(i,j)}-E_{(i,j)})^{2}. \end{aligned}$$

In this context, $P_{(i,j)}$ represents the pixel values of the original image, and $E_{(i,j)}$ represents the pixel values of the encrypted image at the coordinates (*i*, *j*). Both images share the same dimensions, $M \times N$. The MSE serves as a metric to quantify the extent of change induced by the encryption on the image. A higher MSE value indicates less similarity between the original and encrypted images, which is desirable as it suggests more effective encryption. The MSE values obtained through the proposed MIE algorithm, along with those reported in contemporary studies, are illustrated in Table 5. This comparison highlights the effectiveness of the encryption in altering the visual data significantly compared to the original image.

**Table 5:** Comparison of MSE values with other algorithms from the literature.

Images	Proposed	^27^	^44^	^67^	^68^	^69^
House	8336.25	8395.53	−	−	−	−
Peppers	10080.2	10065.4	−	7274.44	10151	10, 092.3
Mandrill	8315.03	8320.41	10930.33	6399.05	8609	8295.21
Sailboat	10051.4	10071.9	−	−	−	−
Satellite Image 4	7616.4	−	−	−	−	−
Satellite Image 5	9076.37	−	−	−	−	−

### Peak signal to noise ratio

In particular, the PSNR is closely linked to the MSE and serves as a metric that evaluates the maximum potential error between the original and encrypted images relative to the highest pixel value possible. The PSNR offers an assessment of encryption effectiveness by measuring the extent to which the encrypted image diverges from the original, taking into account the maximum pixel value of the image. The formula for computing PSNR is:

17 $$\begin{aligned} PSNR=10 \log _{10}\Big (\frac{I^{2}_{max}}{MSE}\Big ). \end{aligned}$$

In this context, $I_{\max }$ denotes the highest pixel value achievable for a grayscale image, which is established at 255 for the monochromatic images. The PSNR calculation is based on the inverse of the MSE, suggesting that a lower PSNR is indicative of a more effective encryption method. The PSNR metrics, as determined by the application of the proposed encryption technique on a variety of images and compared with data from the literature, are displayed in Table 6. The data in Table 6 show that the performance of the proposed MIE algorithm is on par with or occasionally exceeds that of its competitors.

**Table 6:** Comparison of PSNR values with other algorithms from the literature.

Images	Proposed	^27^	^44^	^67^	^68^	^69^
House	8.9211	8.89032	−	−	−	−
Peppers	8.09611	8.10248	−	9.55	−	8.09089
Mandrill	8.93217	8.92936	7.7447	10.10	−	8.94253
Sailboat	8.10856	8.0997	−	−	−	−
Satellite Image 4	9.31331	−	−	−	−	−
Satellite Image 5	8.55168	−	−	−	−	−

### Mean absolute error

In effect, the MAE is a crucial metric used to verify the resilience of an encryption system against differential attacks. Like the MSE, the MAE involves a pixel-by-pixel comparison between the original and encrypted images, measuring the deviation between them. However, distinct from the MSE, which computes the differences by squaring them, the MAE employs the absolute values of these differences in its calculation, as demonstrated by the following formula:

18 $$\begin{aligned} MAE=\frac{1}{M\times N}\sum _{i=0}^{M-1}\sum _{j=0}^{N-1}|P_{(i,j)}-E_{(i,j)} |, \end{aligned}$$

where $P_{(i,j)}$ represents the pixel values of the plain image, and $E_{(i,j)}$ represents the pixel values of the encrypted image, spanning the image dimensions of $M \times N$.

Similarly to the MSE, a higher MAE value signifies a greater discrepancy between the original and encrypted images, indicative of a more robust encryption system. Table 7 presents the MAE values for the same images previously examined in Section “Mean squared error” and Section “Peak signal to noise ratio”, along with MAE metrics from comparable research. These findings further validate that the performance of the proposed encryption system is on par with or exceeds that of other documented algorithms.

**Table 7:** Comparison of MAE values with other algorithms from the literature.

Images	Proposed	^27^	^44^	^67^	^68^	^69^
House	75.2181	75.4983	−	−	−	−
Peppers	82.0162	81.9832	−	−	−	81.7740
Mandrill	75.0714	75.1632	92	−	−	75.1659
Sailboat	81.9697	82.1003	−	−	−	−
Satellite Image 4	72.5538	−	−	−	−	−
Satellite Image 5	78.0543	−	−	−	−	−

### Information entropy

Shannon’s Information Entropy (IE) evaluates the degree of randomness across the color channels of an encrypted image. For a grayscale image, the IE is typically calculated using the formula:

19 $$\begin{aligned} H(m)=\sum _{i=1}^{M} p(m_i) \log _2 \frac{1}{p(m_i)}, \end{aligned}$$

where $p(m_i)$ denotes the probability of occurrence of symbol $m_i$ among all *M* possible symbols in the image. A given image’s entropy score quantifies its randomness, designating the average number of bits needed to encode the data of every pixel. An image with no variability would have an entropy score of 0, as it requires no bits for description. Conversely, an image demonstrating complete randomness would necessitate the full range of bits per pixel, that is $2^3 = 8$ bits, representing the theoretical maximum^70^. Although reaching this maximum is practically unachievable, high-quality encryption techniques strive to approximate it closely. Table 8 lists the entropy values achieved by the proposed MIE algorithm introduced in this study. These values generally surpass those reported for other encryption methods in the literature, indicating that the images encrypted by the proposed system display a significant degree of randomness.

**Table 8:** Comparison of entropy values with other algorithms from the literature.

Images	Proposed	^27^	^44^	^67^	^68^	^69^
House	7.99911	7.99729	−	7.9968	−	−
Peppers	7.99917	7.99866	−	7.9973	7.9997	7.99877
Mandrill	7.99914	7.99834	7.9991	7.9968	7.9998	7.99907
Sailboat	7.99906	7.99875	−	−	−	−
Satellite Image 4	7.99892	−	−	−	−	−
Satellite Image 5	7.99906	−	−	−	−	−

### Correlation coefficient analysis

The pixel cross-correlation analysis is conducted to evaluate the local coherence of pixels within an image, specifically examining the similarity of colors between adjacent pixels. The mathematical formula for the pixel correlation coefficient is:

20 $$\begin{aligned} \rho (x,y)= \frac{cov(x,y)}{\sqrt{\sigma (x)}\sqrt{\sigma (y)}}, \end{aligned}$$

where,

21 $$\begin{aligned} cov(x,y)= \frac{1}{N}\sum _{i=1}^{N}(x_i-\mu (x))(y_i-\mu (y)), \end{aligned}$$

22 $$\begin{aligned} \sigma (x) = \frac{1}{N}\sum _{i=1}^{N}(x_i-\mu (x))^2, \end{aligned}$$

23 $$\begin{aligned} \mu (x)= \frac{1}{N}\sum _{i=1}^{N}(x_i). \end{aligned}$$

In an ideal encryption scenario, the $\rho (x,y)$ values for any pair of pixels (*x*, *y*) should be close to 0, demonstrating the absence of correlation between the original and encrypted images. Conversely, a high $\rho (x,y)$ value would suggest a less effective encryption algorithm, as it indicates a strong similarity between the original and encrypted images. To comprehensively assess the performance of an encryption algorithm, the pixel cross-correlation coefficient is calculated for all adjacent pixel pairs, in various directions.

**Fig. 14:**
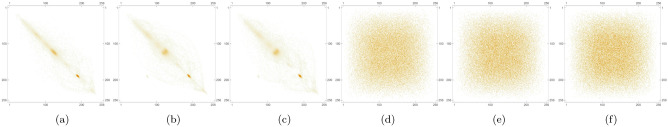
2D Visual representations of co-occurrence matrices of the House image pre- and post-encryption.

**Fig. 15:**
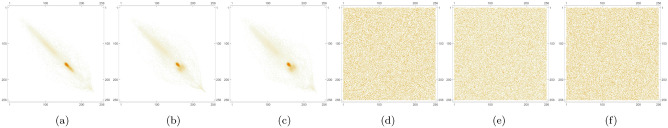
2D Visual representations of co-occurrence matrices of the **red** color channel of House image pre- and post-encryption.

**Fig. 16:**
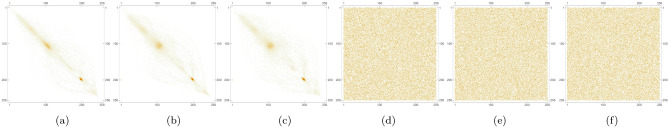
2D Visual representations of co-occurrence matrices of the **green** color channel of House image pre- and post-encryption.

**Fig. 17:**
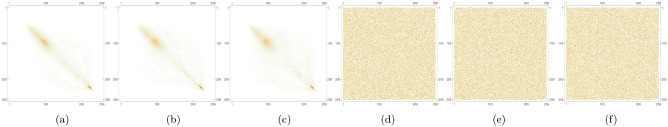
2D Visual representations of co-occurrence matrices of the **blue** color channel of House image pre- and post-encryption.

Table 9 presents the cross-correlation values for plain and encrypted images processed using the proposed MIE algorithm, while Table 10 contrasts these findings with those from recent studies. The encrypted images exhibit cross-correlation values that are nearly zero, signifying a substantial decrease in pixel correlation as a result of the encryption, in contrast to the high correlation seen in the plain images. This reduction is further illustrated in Figs. 14, 15, 16, and 17, which show 2D visualizations of the cross-correlation matrices for a standard ’House’ image and its RGB channels, respectively. These 2D plots display the significant contrast in correlation before and after encryption, underscoring the effectiveness of the proposed encryption method.

**Table 9:** Comparison between correlation coefficients of plain and encrypted images.

	Plain image			Encrypted image		
Image	Horizontal	Diagonal	Vertical	Horizontal	Diagonal	Vertical
House	0.978232	0.936044	0.952926	$-0.00392847$	$-0.00247355$	$-0.0066417$
Peppers	0.959422	0.930426	0.966795	0.00532219	$-0.000599774$	0.00445526
Mandrill	0.848778	0.750624	0.79088	0.000208593	$-0.00393718$	0.000522149
Sailboat	0.952381	0.919872	0.950138	0.00081823	0.00371487	$-0.00346439$
Satellite Image 4	0.975433	0.936835	0.964613	$-0.0037992$	$-0.00479489$	0.00632025
Satellite Image 5	0.886065	0.750058	0.843695	$-0.00423551$	$-0.00331787$	0.00217491

**Table 10:** Comparison of coefficients of the encrypted House image among various schemes from the literature.

Scheme	Horizontal	Diagonal	Vertical
Proposed	$-0.00392847$	$-0.00247355$	$-0.0066417$
^27^	0.0064113	$-0.0015143$	0.000568333
^71^	0.0007832	$-0.0028532$	$-0.0018442$
^72^	0.0005	0.0032	0.0014
^73^	$-0.0107$	0.00067	$-0.027067$

### Discrete Fourier transformation analysis

The Discrete Fourier Transform (DFT) is a pivotal analytical tool utilized to assess the diffusion of content within an encrypted image. By transitioning signals from the time domain to the frequency domain, the DFT deconstructs them into their sinusoidal components. This process is equally applicable to images, facilitating the examination of an image’s frequency spectrum both before and after encryption to identify any residual patterns or artifacts. For an image with dimensions $N \times N$ and pixel values *f*(*i*, *j*), the DFT is mathematically expressed as follows:

24 $$\begin{aligned} F(k,l)=\sum _{i=0}^{N-1} \sum _{j=0}^{N-1} f(i,j) e^{-i 2\pi (\frac{k i}{N}+\frac{l j}{N})}. \end{aligned}$$

In this equation, $f(i, j)$ denotes the pixel intensities in the spatial domain, while $F(k, l)$ stands for the coefficients in the frequency domain. The exponential term serves as the basis function, linking each point $F(k, l)$ in the frequency domain to its corresponding spatial domain counterpart. These basis functions are composed of trigonometric waves, with frequencies that escalate with increasing $k$ and $l$. The component $F(0, 0)$, also known as the DC component, reflects the average brightness of the image. Conversely, $F(N-1, N-1)$ pertains to the highest frequency details present within the image, capturing the finest textures and edges.

**Fig. 18:**
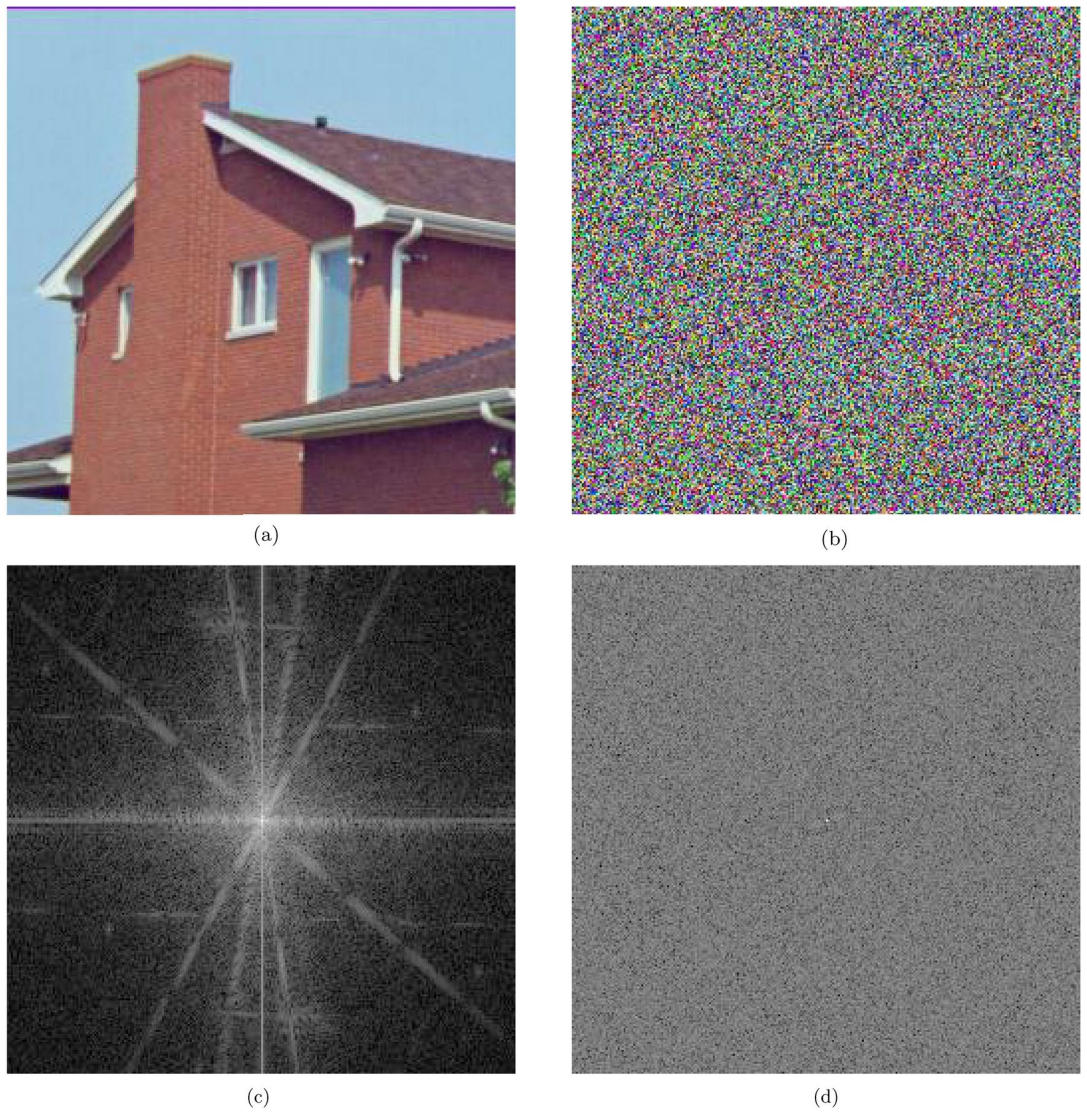
House image and DFT comparison pre- and post-encryption.

Figure 18 visually demonstrates the effects of applying the DFT to both the plain and encrypted versions of the House image. The DFT of the plain image, shown in Fig. 18c, exhibits distinct linear patterns forming a cross-shaped configuration. These patterns underscore the concentration of specific frequencies, reflecting regularities in pixel brightness and indicating the presence of coherent structures and brightness similarities typical of natural images. Conversely, the DFT of the encrypted image, depicted in Fig. 18d, lacks these distinct patterns, instead showcasing a uniform distribution of frequencies across the spectrum. This homogeneity indicates that the original identifiable features have been effectively obliterated by the encryption process, rendering any original patterns unrecognizable.

### Differential attack analysis

A differential attack analysis employs two metrics: the Number of Pixels Change Rate (NPCR) and the Unified Average Changing Intensity (UACI), which both gauge the impact of altering a single pixel in the original image on the encrypted output^74^.

The NPCR specifically measures the percentage of pixel values that alter in the encrypted image following a one-pixel modification in the original image. The mathematical expression for NPCR is given by:

25 $$\begin{aligned} NPCR = \frac{1}{MN} \sum _{i=1}^{M}\sum _{j=1}^{N} D(i,j) \times 100\%, \end{aligned}$$

where *D*(*i*, *j*) is a binary function defined by:

26 $$\begin{aligned} D(i,j) = {\left\{ \begin{array}{ll} 1, & \text {if } I_e^1(i,j) \ne I_e^2(i,j)\\ 0, & \text {otherwise}. \end{array}\right. } \end{aligned}$$

On the other hand, the UACI measures the average intensity of the differences between two encrypted images and is expressed as:

27 $$\begin{aligned} UACI = \frac{1}{M\times N} \sum _{i=1}^{M}\sum _{j=1}^{N} \frac{|I_e^1(i,j) - I_e^2(i,j)|}{255} \times 100\%. \end{aligned}$$

In these formulas, $M$ and $N$ signify the dimensions of the images, whereas $I_e^1(i, j)$ and $I_e^2(i, j)$ indicate the pixel values at the coordinates $(i, j)$ in the first and second encrypted images, respectively.

Table 11 displays the NPCR and UACI metrics derived from the proposed MIE algorithm when applied to different images, demonstrating a high sensitivity to input changes and thus providing robust defense against differential attacks. Additionally, Table 12 offers a comparative analysis with similar metrics from recent studies, underscoring the competitive edge of the proposed MIE algorithm. The NPCR and UACI values achieved fall within the optimal range, underscoring the effectiveness of the proposed MIE algorithm in defending against differential attacks and confirming its suitability as a secure method for image encryption tasks.

**Table 11:** NPCR and UACI for various images.

Metric	Image	Result
NPCR	House	99.6078
	Peppers	99.6119
	Mandrill	99.5967
	Sailboat	99.6201
	Satellite Image 4	99.6023
	Satellite Image 5	99.6068
	Average	99.6076
UACI	House	29.4973
	Peppers	32.1632
	Mandrill	29.4397
	Sailboat	32.145
	Satellite Image 4	28.4525
	Satellite Image 5	30.6095
	Average	30.6095

**Table 12:** Comparison of the NPCR and UACI values computed for various images’ color channels.

Metric	Image	Color channel	Proposed	^75^	^69^	^76^
NPCR	Peppers	Red	99.588	99.6307	99.6032	99.6032
		Green	99.6017	99.6250	99.6032	99.6261
		Blue	99.646	99.6213	99.3750	99.5987
	Mandrill	Red	99.5834	99.6102	99.5880	N/A
		Green	99.6033	99.6134	99.5880	N/A
		Blue	99.6033	99.6057	99.5880	N/A
UACI	Peppers	Red	28.8918	33.4832	33.3459	33.5501
		Green	33.8141	33.4904	33.4702	33.5548
		Blue	33.7838	33.4619	33.4357	33.3849
	Mandrill	Red	29.58	33.5002	33.4273	N/A
		Green	28.0416	33.4711	33.4635	N/A
		Blue	30.6976	33.4951	33.7951	N/A

### Histogram dependency tests

To evaluate the correlation between the original and encrypted images, a linear dependency test is conducted by analyzing the histograms pre- and post-encryption. An optimal encryption approach strives for a dependency coefficient of 0, which signifies no correlation. Conversely, coefficients of 1 or $-1$ suggest strong positive or negative correlations, respectively. This investigation implements five distinct methods for assessing linear correlation: Blomqvist’s $\beta$, Goodman-Kruskal’s $\gamma$, Kendall’s $\tau$, Spearman’s $\rho$, and Pearson’s correlation coefficient *r*.

Within the realm of central tendencies, Blomqvist’s measure quantifies the correlation between two histogram distributions, labeled *X* and *Y*, by leveraging their median values *x* and *y*. This correlation, known as the medial correlation coefficient, is determined by the discrepancy between the probability that the products $(X - x)$ and $(Y - y)$ yield positive results and the probability of them yielding negative outcomes. This calculation is encapsulated in the equation:

28 $$\begin{aligned} \beta = P{(X-\overline{x})(Y-\overline{y})>0} - P{(X-\overline{x})(Y-\overline{y})<0}. \end{aligned}$$

The Goodman–Kruskal metric evaluates monotonic relationships by scrutinizing the sequential arrangement of elements within pairs of histograms. This method hinges on tallying occurrences where elements from both distributions simultaneously increase or decrease, thus influencing linear correlation. The final evaluation derives from the counts of these two types of pairs, designated as $n_c$ (concordant) and $n_d$ (discordant). The Goodman–Kruskal correlation coefficient is thus defined by the following ratio:

29 $$\begin{aligned} \gamma = \frac{n_c - n_d}{n_c + n_d}. \end{aligned}$$

The assessment of Kendall’s correlation considers the sample size and contrasts this with the number of concordant and discordant pairs. The correlation coefficient, which quantifies the degree of association between the datasets, is computed using the following formula:

30 $$\begin{aligned} \tau = \frac{n_c - n_d}{\frac{n(n - 1)}{2}}. \end{aligned}$$

Spearman’s correlation assesses the rank correlation by comparing the rank positions of elements within the histogram to the average rank. This method focuses on the relationship between the rankings of the data rather than the data values themselves. The correlation coefficient, which quantifies the degree of association based on rank data, is expressed using the following formula:

31 $$\begin{aligned} \rho = \frac{\sum (R_{ix} - \overline{R}x)(R{iy} - \overline{R}y)}{\sqrt{\sum (R{ix} - \overline{R}x)^2\sum (R{iy} - \overline{R}_y)^2}}. \end{aligned}$$

Pearson’s correlation, a widely utilized metric, evaluates the linear relationship between distribution values and their mean averages. This correlation coefficient measures the strength and direction of a linear relationship between two variables. It is calculated through the formula:

32 $$\begin{aligned} r = \frac{\sum (X_{i} - \overline{X})(Y_{i} - \overline{Y})}{\sqrt{\sum (X_{i} - \overline{X})^2\sum (Y_{i} - \overline{Y})^2}}. \end{aligned}$$

Table 13 presents the outcomes of these five correlation tests for various test images. The scores, which are all near 0, indicate a substantial absence of correlation between the original and encrypted images, as evident across the histograms of all color channels.

**Table 13:** Histogram dependency tests for various images.

Image	Color	$\beta$	$\gamma$	$\tau$	*r*	$\rho$
House	Red	$-0.0793751$	$-0.0266021$	$-0.0258352$	$-0.0384637$	$-0.0330798$
	Green	$-0.0117881$	$-0.0295528$	$-0.0292214$	$-0.0460022$	0.0174038
	Blue	0.0433977	0.0433977	0.0433977	0.026835	0.019177
	Combined	$-0.03125$	$-0.0759635$	$-0.0754802$	$-0.113166$	$-0.106695$
Peppers	Red	$-0.0118815$	0.0284542	0.0278246	0.0427044	0.0315671
	Green	$-0.111579$	$-0.0612526$	$-0.0605749$	$-0.0648959$	$-0.0894927$
	Blue	$-0.0395334$	0.0339985	0.0334615	0.0285761	0.0483794
	Combined	0.0470592	0.0513736	0.0510389	0.0821866	0.0782085
Mandrill	Red	0.031754	0.0266224	0.0263311	0.0369217	$-0.00184149$
	Green	$-0.111125$	$-0.0285206$	$-0.0279967$	$-0.0421113$	$-0.0398004$
	Blue	0.0478426	0.0480658	0.047575	0.0720131	0.0443447
	Combined	$-0.0118824$	$-0.012257$	$-0.0121945$	$-0.0214346$	$-0.0257524$
Satellite Image 4	Red	0.0635081	0.00990099	0.00980635	0.0340765	0.0193151
	Green	0.0512849	0.029206	0.00874576	0.026184	0.0414366
	Blue	0.0749549	0.0113047	0.0111972	0.009120784	0.0169569
	Combined	$-0.0594158$	$-0.0193528$	$-0.0192502$	$-0.0319917$	$-0.0269471$
Satellite Image 5	Red	0.102369	0.0813297	0.0799339	0.117345	0.0992417
	Green	0.0485914	0.0454316	0.044739	0.0648395	0.0473295
	Blue	0.064008	0.0394213	0.0388268	0.0555381	0.0250862
	Combined	0.196863	0.09316	0.0924558	0.138657	0.0405305

### Key space analysis

The key space in cryptographic systems represents the entire set of potential keys that an encryption algorithm can use. An expansive key space enhances the security of the encryption method by complicating brute-force attacks, due to the significantly high number of keys that need to be tested. The key space should be sufficiently large to deter any feasible key-guessing attempts, yet it should remain practical for key generation, distribution, and storage. The configuration of the key space is crucial as it directly influences the overall strength and security of the cryptographic system.

The proposed MIE algorithm is constructed through the sequential combination of four stages. The variables comprising the key in each of the stages are described as follows:

1. First Stage: To generate a sequence form the memristor system, 15 variables are input into the system. Since 3 sequences are required, one for each channel, a total of 45 control variables are necessary.
2. Second Stage: To generate a sequence form the memristor system, 15 variables are input into the system. Since 3 sequences are required, one for each channel, a total of 45 control variables are necessary.
3. Third Stage: To generate a sequence form the 6D hyperchaotic system, 12 variables are input into the system. Since 3 sequences are required, one for each channel, a total of 36 control variables are necessary.
4. Fourth Stage: To generate two sequences form the 6D hyperchaotic system, 24 variables are input into the system. Since 3 sequences are required, one for each channel, a total of 72 control variables are necessary.

There is a total of $45+45+36+72=198$ variables, where each variable is considered as a real value for brute force computation, and with a maximum machine precision of $10^{-16}$, the key space is computed to be $10^{198 \times 16} = 10^{3168}$, which approximates to $2^{10524}$.

**Table 14:** Key space of various algorithms from the literature.

Algorithm	Key space
Proposed	$2^{10524}$
^2^	$2^{260}$
^26^	$2^{744}$
^32^	$2^{1754}$
^37^	$2^{200}$
^69^	$2^{425}$
^77^	$2^{256}$

Table 14 presents key space sizes for various encryption algorithms, highlighting significant differences. The proposed MIE algorithm features an exceptionally large key space of $2^{10524}$, significantly surpassing others like $2^{744}$ from^26^ and $2^{425}$ from^69^. Such a vast key space indicates a potentially higher level of security, suggesting robust resistance to brute-force attacks. In contrast, the smallest listed key space from^77^ is $2^{256}$, which, while secure, is modest compared to the proposed MIE algorithm. This comparison underscores the critical role of large key spaces in enhancing cryptographic security.

### Time and complexity analyses

An analysis of execution time is crucial for assessing the efficiency and suitability of an encryption algorithm for real-time use. Table 15 shows the average execution times of the proposed image encryption method, computed for the mean of 50 iterations for various image sizes. When used on mobile devices, it takes less than a quarter of a second to encrypt an image that measures $256 \times 256$ pixels.

Table 16 shows the runtime using different machines and compares those times with the literature. Machine A needs less than one eighth of a second, while machine B needs about one quarter of a second, for an image of dimensions $256 \times 256$ pixels. For comparable machine specifications, the proposed MIE algorithm is shown to provide a comparable or superior runtime performance in relation to its counterparts from the literature. It is important to recognize that the reported and measured encryption times of different algorithms are influenced not only by the proposed MIE algorithm’s inherent complexity but also by factors such as the machine’s processing power, RAM availability, the software package or programming language used, among other considerations.

In the analysis of the time complexity for the proposed image encryption scheme, it is important to note that, like many traditional encryption algorithms, it operates in *O*(*n*) time complexity, where *n* is the number of bits in the image. This linear time complexity ensures that the encryption time scales directly with the size of the input.

**Table 15:** Encryption time of augmented images for $K=4$, at varying dimensions of $M\times M$.

Augmented image dimensions	Time [s]
$64 \times 64$	0.0174975
$128 \times 128$	0.0736345
$256 \times 256$	0.230717
$512\times 512$	0.960692
$1024\times 1024$	3.79707

**Table 16:** Encryption time analysis comparison of encrypting an image of dimensions $256 \times 256$.

Scheme	Time [s]	Computer Specs.	Time Complexity
Proposed, Machine A	0.148071	Intel^®^ Core^TM^ i9 @ 2.9 GHz, 32 GB	*O*(*n*)
Proposed, Machine B	0.230717	Intel^®^ Core^TM^ i$7-7500$U CPU @ 2.70GHz, 8GB	*O*(*n*)
^23^	0.2194	Intel^®^ Core^TM^ i$7-1195G7$U CPU @ 2.90GHz, 32GB	*O*(*n*)
^69^	2.582389	Intel^®^ Core^TM^ i7 processor , 2400 MHz and 32 GB	*O*(*n*)
^77^	0.473	Intel^®^ Core^TM^ i$7-6700$ CPU @ 3.40 GHz, 8 GB.	*O*(*n*)

### The National institute of standards and technology analysis

The U.S. National Institute of Standards and Technology (NIST) offers a range of statistical tools and resources for cryptography. One of these resources is the SP 800-22 statistical test suite, which includes various tests and analyses designed to assess the performance of PRNGs. This suite is particularly valuable for evaluating the output of encryption algorithms. Although it is not specifically tailored to gauge the strength of an encryption scheme, data that successfully passes the tests in the NIST SP 800-22 suite exhibits sufficient randomness to be deemed secure. As illustrated in Table 17, an encrypted bit-stream generated by the proposed MIE algorithm passes all the tests in the suite, with all values surpassing the minimum acceptance threshold of 0.01. This result indicates that the encryption produced by the proposed MIE algorithm is robust enough to function effectively as a PRNG.

**Table 17:** Results of the NIST test suite.

Test	*p*-value	Result
Frequency	0.908588	Success
Block Frequency	0.099121	Success
Run	0.546632	Success
Long runs of ones	0.778769	Success
Rank	0.810812	Success
Spectral F.F.T.	0.013013	Success
Non overlapping	0.117835	Success
Overlapping	0.667857	Success
Universal	0.571256	Success
Serial	0.350226	Success
Serial	0.308997	Success
Approx. entropy	0.509617	Success
Cum. sums forward	0.694468	Success
Cum. sums reverse	0.801355	Success
Random Excursions (R.E.) 1	0.983382	Success
R.E. 2	0.912714	Success
R.E. 3	0.753009	Success
R.E. 4	0.091431	Success
R.E. 5	0.631733	Success
R.E. 6	0.896141	Success
R.E. 7	0.480969	Success
R.E. 8	0.910560	Success
Random Excursions Variant (R.E.V.) 1	0.831025	Success
R.E.V. 2	0.915573	Success
R.E.V. 3	0.692215	Success
R.E.V. 4	0.671244	Success
R.E.V. 5	0.958419	Success
R.E.V. 6	0.673596	Success
R.E.V. 7	0.930324	Success
R.E.V. 8	0.972985	Success
R.E.V. 9	0.225437	Success
R.E.V. 10	0.491092	Success

### S-box analysis

The robustness of S-boxes is vital for the security of cryptographic systems. In this study, three novel S-box designs are proposed, based on sequences generated from the modified BBS algorithm. To quantitatively evaluate these S-boxes, a series of established cryptographic metrics have been employed, as in^49^. Each of these metrics is described as follows:

- Non-Linearity (NL): The NL quantifies how much an S-box’s output diverges from any linear or affine function. Higher NL values suggest stronger resistance to linear cryptanalysis, which is essential for secure S-box designs.
- Linear Approximation Probability (LAP): The LAP processes the likelihood of efficaciously estimating the S-box function through linear expressions. S-boxes with lower LAP values are deemed more secure, as they demonstrate decreased vulnerability to linear cryptanalytic attacks.
- Differential Approximation Probability (DAP): The DAP quantifies the probability that specific input differentials will result in particular output differentials. To safeguard against differential cryptanalysis, S-boxes are designed to exhibit a low DAP.
- Bit Independence Criterion (BIC): The BIC evaluates the dependency of output bits on variations in input bits. For an S-box to be resistant against harsh cryptanalysis, any variation in an input bit should result in random changes in the output bits.
- Strict Avalanche Criterion (SAC): The SAC assesses the impact of variations in input bits on changes in output bits. A given S-box adhering to the SAC guarantees that each output bit exhibits a 0.5 likelihood of changing when a single input bit is flipped, thereby increasing the system randomness.

The values calculated for these metrics for the three proposed S-box designs are presented in Table 18. This table provides a comparative investigation with S-boxes from existing literature, showing that the performance of the S-boxes proposed meets or outperforms that of current S-boxes in these key metrics. Additionally, the data in Table 18 validate the robustness of the proposed S-box designs, establishing them as essential elements in the security of the overall proposed MIE algorithm. High performance in these metrics ensures that the proposed S-boxes offer strong protection against various cryptanalytic attacks, thereby securing the image encryption process. Beyond these metrics, it is also important to consider the construction of S-Boxes without fixed-points, reverse fixed-points or short period rings^78–80^.

**Table 18:** Comparison among the proposed S-boxes and those in the literature.

S-box	NL	SAC	BIC	LAP	DAP
Ideal values	112	0.5	112	0.0625	0.015625
Prop. S-box1	108	0.506836	100	0.109375	0.015625
Prop. S-box2	108	0.499756	108	0.078125	0.015625
Prop. S-box3	108	0.498047	100	0.109375	0.015625
^27^ MT	108	0.503662	92	0.140625	0.015625
^27^ OSSL	108	0.499023	112	0.0625	0.015625
^27^ IMKL	108	0.499268	104	0.09375	0.015625
^26^	106	0.47266	68	0.23438	0.015625
^30^ SNM	106	0.499268	104	0.09375	0.015625
^30^ HC 4D	108	0.500977	108	0.078125	0.015625
^30^ HC 7D	108	0.506592	108	0.078125	0.015625
^81^	112	0.4998	112	0.0625	0.0156
^82^	107	0.497	103.5	0.1560	0.039

## Conclusions and suggested future research

This study introduced an innovative MIE algorithm specifically designed to bolster the security of satellite imagery. The proposed MIE algorithm incorporated a sophisticated blend of hyperchaotic systems, SVD, RC5 encryption in counter mode, a chaotic-based Hill cipher, and a custom S-box created using a modified BBS algorithm. Extensive numerical testing validated the proposed MIE algorithm’s robust resistance against various cryptographic attacks, including statistical, differential, and brute-force methods, thereby confirming its effectiveness in safeguarding the integrity, confidentiality, and security of image data. Moreover, the proposed MIE algorithm is designed to operate efficiently, enabling real-time encryption capabilities that are crucial for handling the high throughput and real-time processing demands of satellite imagery. One notable limitation identified in the proposed MIE algorithm is its utilization of the numerical solutions of two hyperchaotic continuous systems of differential equations. Clearly, the use of discrete chaotic maps would result in an even higher efficiency.

Future work could focus on the implementation of this MIE algorithm on a Field-Programmable Gate Array (FPGA). This would enable a thorough examination of the proposed MIE algorithm’s performance in a hardware setting, potentially unlocking faster processing speeds and encryption capabilities. This exploration aimes to optimize the algorithm’s efficiency and practicality, making it more suitable for high-demand security applications where speed and reliability are paramount. Furthermore, the consideration of cryptanalysis research targeted at image encryption algorithms holds significant guidance during the design phase of image encryption algorithms^83^. From such research, it is clear that the generation and utilization of dynamic or one-time keys would prove beneficial in countring key leakage attacks^84^.

## Data Availability

The datasets used and/or analyzed during the current study are available from the corresponding author on reasonable request.
